# Comparative deep learning approaches for bean leaf disease recognition

**DOI:** 10.3389/fpls.2026.1703449

**Published:** 2026-04-29

**Authors:** Kollipara Anirudh, Darbha Srujan, Aditya Sai, Gnana Swathika O. V.

**Affiliations:** 1 School of Electrical Engineering, Vellore Institute of Technology, Chennai, India; 2 Centre for Smart Grid Technologies, Vellore Institute of Technology, Chennai, India

**Keywords:** bean leaf disease detection, computer vision, convolutional neural network (CNN), deep learning, image recognition, image classification, precision agriculture, ResNet18

## Abstract

**Context:**

Plant diseases are a serious danger to the world's food security since they drastically lower crop output. Traditional manual plant leaf inspection is time-consuming, labor-intensive, and frequently subjective. Recent developments in deep learning provide effective and scalable methods for image-based analysis-based automated plant disease identification.

**Techniques:**

Three deep learning architectures—a proprietary Convolutional Neural Network (CNN), ResNet18, and Vision Transformer (ViT)—are used in this study to examine automated bean leaf disease identification. The Augmented iBean dataset, which has three classes—angular leaf spot, bean rust, and healthy leaves—was used to train and assess the models. Every model was trained using the same preprocessing and training settings to provide fair benchmarking. Receiver Operating Characteristic (ROC) curves, accuracy, precision, and confusion matrices were used to assess the model's performance.

**Outcomes:**

ResNet18 fared better than CNN and Vision Transformer models, according to a comparative analysis. ResNet18 maintained a high level of computing efficiency while achieving 99% accuracy and 99.01% precision. Its better categorisation capacity across all disease categories was validated using confusion matrix and ROC analysis.

**In conclusion:**

The study shows that ResNet18 offers the optimal trade-off between accuracy and efficiency and creates a standard benchmarking framework for bean leaf disease identification. The results demonstrate its applicability for real-time deployment in precision agricultural systems for better crop management and early disease identification.

## 1 Introduction

Agriculture is the foundation of food security globally, providing nutrients to billions of people, and continues to provide national economic stability for billions of people worldwide. The global population is projected to increase to over 9.7 billion by 2050 and may stress agricultural systems worldwide to increase productivity while ensuring sustainability and resilience. Plant diseases pose one of the most severe threats to agricultural productivity, responsible for annual yield losses ranging from 20% to 40% worldwide (Adewusi et al., 2024). Among these, leaf-based plant diseases are particularly critical because they manifest through visible lesions, discoloration, and deformities that hinder photosynthesis and overall plant health (Rahul et al., 2020). Early detection of these diseases is therefore essential to implement effective management strategies and minimize crop losses.

Conventional methods for disease identification in agriculture typically involves manual inspection by farmers which often is labor-intensive, time-consuming, and impractical for large-scale deployment in resource-constrained environments (Tewari et al., 2020). If the disease is not identified in the early stages, there may be a significant production loss to the farmer. One example is with bean crops that suffer from angular leaf spot and bean rust, where in the past, these diseases have been responsible for large epidemics that threaten food supplies in developing and developed countries. Beans are one of the most important legume crops that are grown around the world. Beans are important as a nutritional element for many people and are important as a source of protein, dietary fiber, and essential minerals for millions of people. It is very important to control diseases in bean crops, especially in parts of the world where beans are a staple diet, to ensure food security.

In recent years, Artificial Intelligence (AI) and Deep Learning (DL) have emerged as transformative tools for agricultural innovation, particularly in plant-disease recognition, yield prediction, and resource optimization (Vikas et al., 2021). AI-driven systems leverage image processing and classification to automate the detection of diseases from leaf imagery, significantly reducing the need for manual analysis (Ramakrishnam Raju et al., 2022). Combined with Internet of Things (IoT) and mobile-based technologies, AI now enables real-time disease monitoring using drones, edge devices, and cloud computing, empowering scalable smart-farming systems (Yépez-Ponce et al., 2023; Widianto et al., 2024).

Several studies have demonstrated the growing integration of robotics, automation, and smart sensors for agricultural operations such as chemical spraying (Tewari et al., 2020), seedling transplantation (Rahul et al., 2020; Vikas et al., 2021), and precision irrigation (Limbo et al., 2025; Alazzai et al., 2024). These technologies have evolved from automation into data-driven decision systems, where AI models continuously optimize inputs such as water, fertilizer, and pesticides to enhance yield and minimize environmental impact (Gayathri et al., 2024; Vaiyapuri and Aldosari, 2025).

The utilization of AI in the field of plant disease detection has matured rapidly with the introduction of deep learning models capable of analyzing large-scale leaf-image datasets. Convolutional Neural Networks (CNNs) have been the backbone of image-based classification due to their hierarchical feature-extraction capability (Mallinger et al., 2024). CNNs have achieved success in identifying diseases in crops such as rice, potato, and maize (Shinde et al., 2024). However, CNNs are inherently limited by their localized receptive fields and are unable to capture long-range spatial dependencies critical for understanding diseases distributed across larger leaf areas (Gryshova et al., 2024).

To overcome these limitations, more advanced architectures have emerged, including Residual Networks (ResNets) and Vision Transformers (ViTs). ResNet18 incorporates skip (residual) connections that mitigate vanishing gradients and allow deeper models to learn complex representations (Berrahal and Berqia, 2024). This structure enables the capture of both local and global image features efficiently, improving robustness in disease classification tasks (Adewusi et al., 2024; Kaur et al., 2024). In contrast, Vision Transformers use self-attention mechanisms to model global contextual relationships across the entire image, thereby enhancing their ability to distinguish subtle disease patterns (Gryshova et al., 2024). These approaches represent a shift toward architectures that balance feature locality and holistic spatial reasoning key for leaf disease diagnosis.

The advancement of these architectures aligns with the broader movement toward explainable and sustainable AI in precision agriculture (Mallinger et al., 2024). Recent research emphasizes the deployment of AI-driven systems integrated with IoT frameworks for tasks such as irrigation management, nutrient optimization, and pest control (Limbo et al., 2025; Alazzai et al., 2024),. Moreover, AI is now central to climate-smart farming strategies, optimizing resource use and mitigating environmental impact (Gryshova et al., 2024; Assimakopoulos et al., 2025). EfficientNet, ConvNeXt, and hybrid CNN Transformer models have further advanced performance while reducing computational cost (Berrahal and Berqia, 2024; Kaur et al., 2024) (Manikandan et al., [[NoYear]]). In addition, explainable and edge-optimized AI methods (Vaiyapuri and Aldosari, 2025; Mallinger et al., 2024; Assimakopoulos et al., 2025), have improved the interpretability and feasibility of deploying DL systems directly on farm-level hardware.

**Figure 1:**
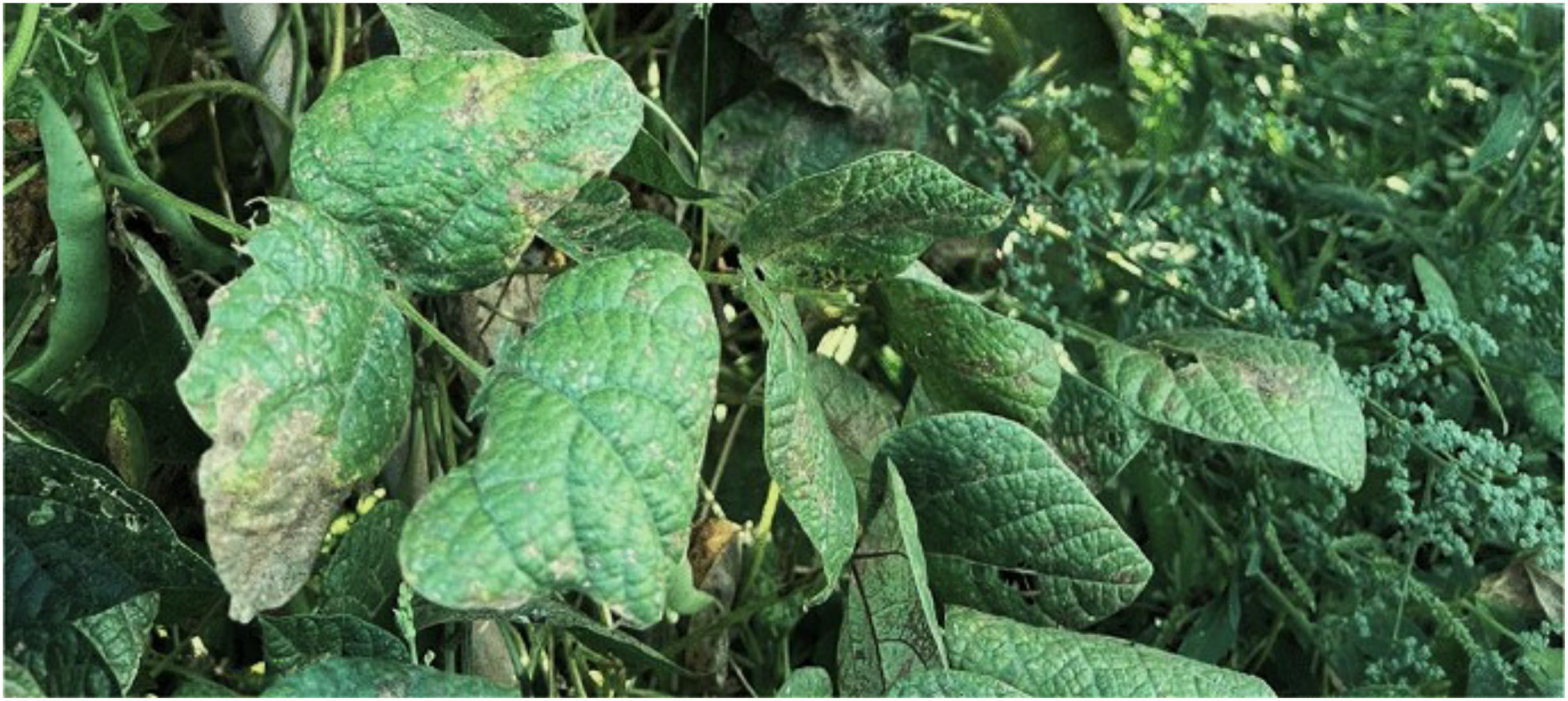
Disease affected bean leaves.

Despite these advances, major challenges remain. Unlike prior studies that report performance in isolation, this work emphasizes controlled benchmarking under identical experimental conditions to ensure fair comparison across architectures. This is critical for identifying models suitable for real-world agricultural deployment where both accuracy and efficiency are equally important. Many prior studies have relied on small or imbalanced datasets, limiting generalization and reproducibility (Adewusi et al., 2024) (Limbo et al., 2025). Moreover, few have compared CNN, ResNet, and ViT architectures under uniform conditions to evaluate their relative trade-offs in accuracy, efficiency, and scalability. The high computational demands of transformer-based models also present barriers to adoption in rural or resource-constrained environments (Alazzai et al., 2024; Akbar et al., 2025). Therefore, identifying models that balance precision, generalization, and efficiency is essential for practical agricultural deployment. Despite extensive research on AI-enabled smart farming systems, a gap remains in systematically benchmarking modern deep learning architectures for plant disease recognition under controlled conditions. This study addresses this gap by positioning CNNs, residual networks, and transformers within a unified experimental framework. The research addresses these challenges by performing a rigorous comparative analysis of three deep learning architectures—CNN, ResNet18, and Vision Transformer—on the Augmented iBean Dataset (Hosny et al., 2023; Hashem et al., 2024; Aboelenin et al., 2025; Alhammad et al., 2025; Hosny et al., 2025). This large and balanced dataset includes over 46,000 images categorized into three classes: angular leaf spot, bean rust, and healthy leaves. By maintaining consistent preprocessing, augmentation, and hyperparameter settings, this study ensures a fair and transparent comparison among the models. Performance is evaluated using multiple metrics, including accuracy, precision, recall, F1-score, and ROC–AUC, to provide a comprehensive assessment of diagnostic performance. Furthermore, we implement our experiments in Python using the Kaggle platform, leveraging GPU acceleration to ensure fair and consistent model training and evaluation. The **Figure 1** shows the image of some bean leaves.

The primary novelty of this research lies in establishing a standardized benchmarking framework under identical experimental conditions, addressing reproducibility gaps in prior comparative studies. Additionally, we provide explicit quantification of performance-efficiency trade-offs, enabling practitioners to select architectures appropriate for their computational constraints.

### 1.1 Related work and research gap

This study presents a standardized comparative evaluation of three advanced deep learning architectures—Convolutional Neural Network (CNN), ResNet18, and Vision Transformer (ViT)—for bean leaf disease recognition, ensuring all models are trained and tested under identical experimental conditions to guarantee fairness and reproducibility. A comprehensive performance analysis is conducted using multiple evaluation metrics, including accuracy, precision, recall, F1-score, and ROC–AUC, to provide a holistic understanding of each model’s diagnostic robustness and reliability. Furthermore, the research examines computational efficiency, model complexity, and real-world feasibility, emphasizing deployment in resource-constrained agricultural environments. Through this analysis, ResNet18 is identified as the most balanced architecture, offering an optimal trade-off between high predictive performance and computational cost, making it highly suitable for real-time implementation in precision-agriculture systems.

Prior research in smart agriculture has largely focused on IoT-based automation, robotic farming systems, and decision-support platforms rather than deep learning–based leaf disease benchmarking. Studies such as Rahul et al (Rahul et al., 2020), Tewari et al (Tewari et al., 2020), and Vikas et al (Vikas et al., 2021). emphasize robotic transplanting and precision spraying, while works by Ramakrishnam Raju et al (Ramakrishnam Raju et al., 2022). and Widianto et al (Widianto et al., 2024). focus on IoT-enabled monitoring and control systems. Although these studies demonstrate the growing role of artificial intelligence in agriculture, they do not address the comparative diagnostic capability of modern deep learning architectures for plant disease recognition.

More recent survey-based studies (Adewusi et al., 2024; Gryshova et al., 2024; Kaur et al., 2024; Assimakopoulos et al., 2025; Limbo et al., 2025) comprehensively review AI applications in precision agriculture but primarily discuss algorithmic categories without providing controlled experimental benchmarking. In particular, most reviews report accuracy values in isolation, without analyzing performance–efficiency trade-offs, which are critical for real-world deployment on resource-constrained agricultural hardware.

With respect to plant disease detection, hybrid and transformer-based approaches have begun to emerge. Aboelenin et al (Aboelenin et al., 2025). proposed a hybrid CNN–Vision Transformer framework for plant leaf disease classification, reporting strong accuracy but without isolating the contribution of individual architectures under identical training conditions. Hosny et al (Hosny et al., 2023). explored feature fusion using CNNs and handcrafted descriptors, improving classification performance at the cost of increased computational complexity. Similarly, Alhammad et al (Alhammad et al., 2025). incorporated explainable AI techniques for potato leaf disease classification, focusing on interpretability rather than deployment feasibility.

Recent studies have further extended deep learning approaches for bean and plant leaf disease classification by incorporating architectural enhancements and interpretability mechanisms. Karthik et al (Karthik et al., 2025). proposed an explainable deep learning framework combining a custom CNN with a transformer backbone for bean leaf disease classification. Their work integrates explainability techniques to visualize decision-making regions, thereby improving model transparency. While this approach enhances interpretability, it introduces additional architectural complexity and computational overhead, which may limit deployment on resource-constrained agricultural edge devices.

Similarly, Sebastian et al (Sebastian et al., 2024). introduced ViTaL, an advanced Vision Transformer–based framework that employs linear projection for feature reduction and evaluates its performance against CNN-based architectures on plant leaf datasets. The study demonstrates improved feature efficiency and competitive classification performance; however, it focuses primarily on architectural modification rather than providing a standardized benchmarking of multiple deep learning models under identical experimental conditions.

In contrast to these studies, the present work introduces a standardized benchmarking framework that evaluates CNN, ResNet18, and Vision Transformer models under identical preprocessing, augmentation, and optimization settings on the Augmented iBean dataset. The novelty of this work lies not in proposing a new architecture, but in systematically quantifying accuracy, robustness, and computational efficiency trade-offs, thereby enabling informed model selection for real-world agricultural deployment.

### 1.2 Comparative analysis with existing plant disease identification studies

To address the need for broader contextual comparison, this study situates its findings within existing deep learning–based plant disease identification research. **Table 1** summarizes representative works that report dataset usage, model architecture, architectural contributions, and evaluation metrics.

**Table 1:** Comparison with existing plant disease detection studies.

Study	Dataset	Model used	Architectural focus	Limitation
Ref (Hosny et al., 2023).	PlantVillage	CNN + LBP (Feature Fusion)	Feature fusion	High computational complexity
Ref (Aboelenin et al., 2025).	Mixed crops	Hybrid CNN-ViT	Combined CNN + Transformer features	No isolated benchmarking
Ref (Alhammad et al., 2025).	Potato leaves	CNN + XAI	Explainability (Grad-CAM)	No efficiency analysis
Ref (Karthik et al., 2025).	Bean leaves	Custom CNN + Transformer	Explainable hybrid deep model	Increased architectural complexity
Ref (Sebastian et al., 2024).	Plant leaf datasets	ViT (ViTaL framework)	Transformer + linear projection	No standardized benchmarking
This Work	Augmented iBean	CNN / ResNet18 / ViT	Controlled benchmarking	Balanced accuracy & efficiency

Hosny et al (Hosny et al., 2023). investigated multi-class plant leaf disease classification using convolutional neural networks combined with local binary pattern feature fusion on the PlantVillage dataset. Their approach demonstrated improved discrimination capability over standalone CNNs, albeit with increased architectural complexity.

Alhammad et al (Alhammad et al., 2025). focused on deep learning–based potato leaf disease classification with the integration of explainable AI techniques. Their work highlights the role of interpretability in CNN-based disease detection while maintaining strong classification performance.

Aboelenin et al (Aboelenin et al., 2025). proposed a hybrid CNN–Vision Transformer framework for plant leaf disease detection across mixed crop datasets. By combining convolutional feature extraction with transformer-based global attention, the study achieved high classification effectiveness, though it did not isolate individual architecture performance under uniform training settings.

Karthik et al (Karthik et al., 2025). introduced an explainable deep learning framework combining a custom CNN and transformer components for bean leaf disease classification. Their model emphasizes interpretability alongside competitive performance but does not provide a controlled comparison between classical CNNs, residual networks, and transformers under identical preprocessing conditions.

Sebastian et al (Sebastian et al., 2024). (ViTaL) presented a Vision Transformer–based architecture incorporating linear projection for feature reduction, evaluated on the PlantVillage dataset. The ViTaL framework reports a low Hamming loss, indicating strong classification quality while improving transformer efficiency through dimensionality reduction.

In contrast to prior studies, the present work provides a controlled comparative benchmarking framework in which CNN, ResNet18, and Vision Transformer models are evaluated under identical preprocessing, augmentation, and optimization conditions on the augmented iBean dataset. This enables direct performance and efficiency comparison across architectures, addressing a gap in reproducibility and fairness observed in existing literature. This controlled evaluation eliminates variability arising from dataset differences and training protocols, enabling a more reliable assessment of architectural strengths compared to prior studies.

This paper is organized as follows: Section II describes the methodology, including the dataset, preprocessing, and model architectures. Section III presents the results and discussion, including performance analysis of each model and a comparative evaluation. Section IV provides the conclusion, summarizing the key findings and outlining directions for future work.

## 2 Methodology

### 2.1 Flowchart

**Figure 2:**
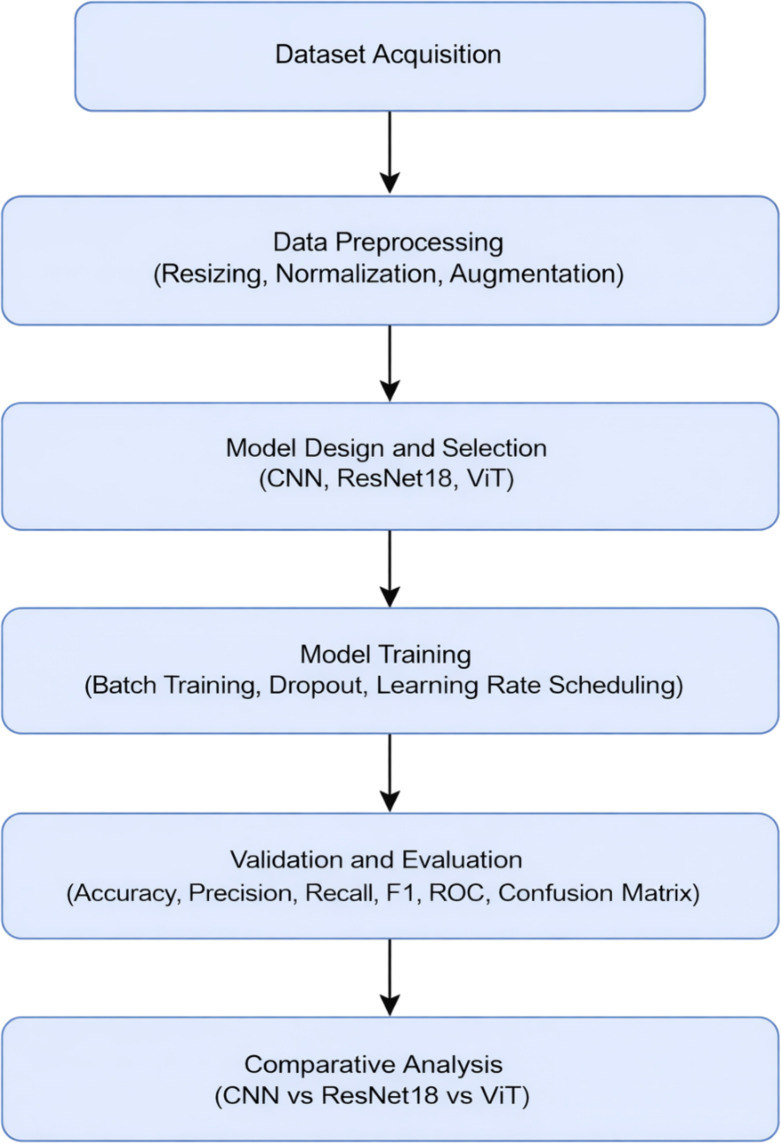
Flowchart for deploying ViT, CNN, ResNet18 for leaf disease detection.

The proposed research follows a structured methodology that begins with dataset collection and preprocessing, followed by the training of three deep learning models (CNN, ResNet18, and Vision Transformer), and finally, performance evaluation. The workflow of the proposed system is illustrated in **Figure 2** and described step-by-step below:

1. Dataset Acquisition: The dataset used in this study is the Augmented iBean Dataset (Hosny et al., 2023; Hashem et al., 2024; Aboelenin et al., 2025; Alhammad et al., 2025; Hosny et al., 2025), which contains over 41,300 bean leaf images across three categories: angular leaf spot, bean rust, and healthy leaves.
2. Data Preprocessing: The dataset undergoes preprocessing, which includes resizing images to a fixed dimension, normalization of pixel values, and augmentation techniques such as rotation, flipping, and contrast adjustment to ensure model robustness. Images were resized to 224×224 pixels. Training images were augmented using random rotation (± 25°), horizontal flip (p = 0.5), brightness & contrast jitter (0.8–1.2 range), Gaussian noise (σ = 0.02), and random perspective transform (0.2). Validation data received only resizing and normalization to avoid data leakage. Pixel values were normalized to [0,1] for CNN and ImageNet mean/std for ResNet18 & ViT.
3. Model Design and Selection: Three deep learning architectures are selected: CNN, ResNet18, and Vision Transformer. Each model is implemented in Python on Kaggle, with appropriate hyperparameters and optimization techniques. Models were implemented using PyTorch with GPU T4 x2 acceleration. CNN was trained from scratch, while ResNet18 and ViT used ImageNet-pretrained weights.
4. Model Training: The training process involves feeding the pre-processed dataset into the models. Standard practices such as batch training, dropout regularization, and learning rate scheduling are applied to improve convergence and avoid overfitting. All models were trained for 10 epochs with early stopping (patience = 5), batch size = 32, learning rate = 1e-4, Adam/AdamW optimizer, cross-entropy loss, and Reduce-LROn-Plateau scheduler (factor = 0.1). Dropout (0.3) and weight decay (1e-4) were used to minimize overfitting. Each experiment was repeated three times, and the mean performance was reported.
5. Validation and Evaluation: A portion of the dataset (5,320 images) is used for validation. The models’ performance is evaluated using metrics such as accuracy, precision, recall, F1-score, confusion matrices, and ROC curves. Evaluation used per-class and macro-averaged metrics to avoid class imbalance bias. ROC-AUC was computed for each class to validate discriminative capability.
6. Comparative Analysis: The results of the three models are compared to determine their relative strengths and weaknesses, with emphasis on their suitability for bean leaf disease recognition.

This systematic methodology ensures that each model is evaluated fairly under identical conditions, providing a reliable comparative analysis. **Figure 2** shows the flowchart used for the research paper.

### 2.2 Architecture selection and motivation

#### 2.2.1 Convolutional neural networks

CNNs excel at extracting localized features through hierarchical convolutional kernels. For bean leaf diseases, CNNs are well-suited for detecting discrete anomalies like rust pustules and angular leaf spots. However, their localized receptive fields limit ability to capture disease patterns distributed across large leaf regions.

This architecture is well tailored for plant disease detection since plant leaf disease is commonly exhibited as localized pattern anomalies (e.g., spots, discolorations) where convolutional kernels can recognize (Adewusi et al., 2024). In this study, a 3-layer CNN with batch normalization, ReLU activation, max-pooling, and dropout (0.3) was used, optimized with Adam and cross-entropy loss. That said, CNNs have intrinsic limitations with respect to modeling global relationships across areas of the image that are spatially distant from each other and are consequently less effective for diseases identified via widespread plant symptoms.

#### 2.2.2 Residual networks (ResNet18)

Residual Networks (ResNets) were introduced to address the vanishing gradient problem in very deep networks. The key innovation is the use of skip connections that allow the gradient to bypass one or more layers during backpropagation (Kaur et al., 2024). Residual connections enable efficient training of deeper networks while capturing multi-scale features. For agricultural deployment, ResNet18 balances high accuracy 99%) with fast inference—critical for edge devices on farms. This architecture captures both fine-grained texture (e.g., pustule morphology) and coarser disease progression patterns.

ResNet18 is a relatively lightweight architecture consisting of 18 layers. It balances depth with computational efficiency, making it particularly suitable for applications requiring high accuracy without excessive computational resources (Gryshova et al., 2024). Consistent with the implementation used in this study, no attention modules were included; instead, the final fully connected layer of ImageNet-pretrained ResNet18 was replaced with a 3-class classifier. Extensive augmentation and ImageNet normalization improved generalization. For agricultural disease detection, ResNet18 is advantageous because it can model both local and global features while maintaining fast training times.

#### 2.2.3 Vision transformer

The Vision Transformer represents a paradigm shift in computer vision. Instead of relying on convolutional kernels, ViTs divide the input image into patches, which are then linearly embedded and processed using self-attention mechanisms (Manikandan et al., [[NoYear]). The self-attention layers enable the model to capture long-range dependencies across the entire image, offering a more holistic understanding of visual data. Self-attention mechanisms enable global image understanding by directly comparing all image regions. ViTs excel at detecting spatially complex disease patterns but require larger datasets and higher compute.

ViTs have demonstrated state-of-the-art performance in various image classification benchmarks (Alazzai et al., 2024). In this study, pretrained ViT-small (patch16-224) was used. Lower transformer layers were frozen, and the classification head and upper blocks were fine-tuned to balance accuracy and computational cost. AdamW optimizer was used with weight decay (0.01). In the case of leaf disease detection, ViTs are particularly beneficial because they can detect both fine-grained local patterns and global disease spread, making them robust against variations in disease presentation. However, ViTs typically require large datasets for effective training, which is why the augmented iBean dataset is highly suitable for this model.

While Vision Transformer achieves strong predictive performance, its higher inference latency and memory footprint limit feasibility for edge deployment. ResNet18 provides the most favorable balance between accuracy and computational efficiency, making it the most practical choice for real-time agricultural applications.

### 2.3 Performance analysis

Evaluating model performance is crucial for determining the effectiveness of deep learning architectures in real-world applications. This study employs several widely recognized performance metrics:

1. Accuracy: Accuracy measures the proportion of correctly classified images out of the total. While it provides an overall sense of model performance, it may be misleading in cases of class imbalance (Akbar et al., 2025).
2. Precision: Precision calculates the proportion of true positive predictions among all positive predictions. In disease detection, high precision indicates that the model makes fewer false disease diagnoses, which is essential for minimizing unnecessary interventions (Hosny et al., 2023; Hashem et al., 2024; Aboelenin et al., 2025; Alhammad et al., 2025; Hosny et al., 2025).
3. Recall: Recall measures the proportion of true positives identified among all actual positives. A high recall ensures that most diseased leaves are detected, which is critical for preventing the spread of disease in crops.
4. F1-Score: The F1-score is the harmonic mean of precision and recall. It provides a balanced measure, especially in cases where both false positives and false negatives carry significant consequences.
5. Confusion Matrix: The confusion matrix provides a detailed breakdown of predictions across different classes, highlighting specific areas where the model may struggle.
6. Receiver Operating Characteristic (ROC) Curve: ROC curves illustrate the trade-off between true positive and false positive rates at various threshold settings. The Area Under the Curve (AUC) provides a single-value summary of the model’s discriminative ability.

By employing this set of metrics, the study ensures a comprehensive evaluation of the three models. Results were averaged across three independent runs to ensure statistical reliability and minimize variance. Thus, it allows for robust conclusions regarding their suitability for bean leaf disease detection in precision agriculture.

## 3 Results and discussion

### 3.1 Dataset description

**Figure 3:**
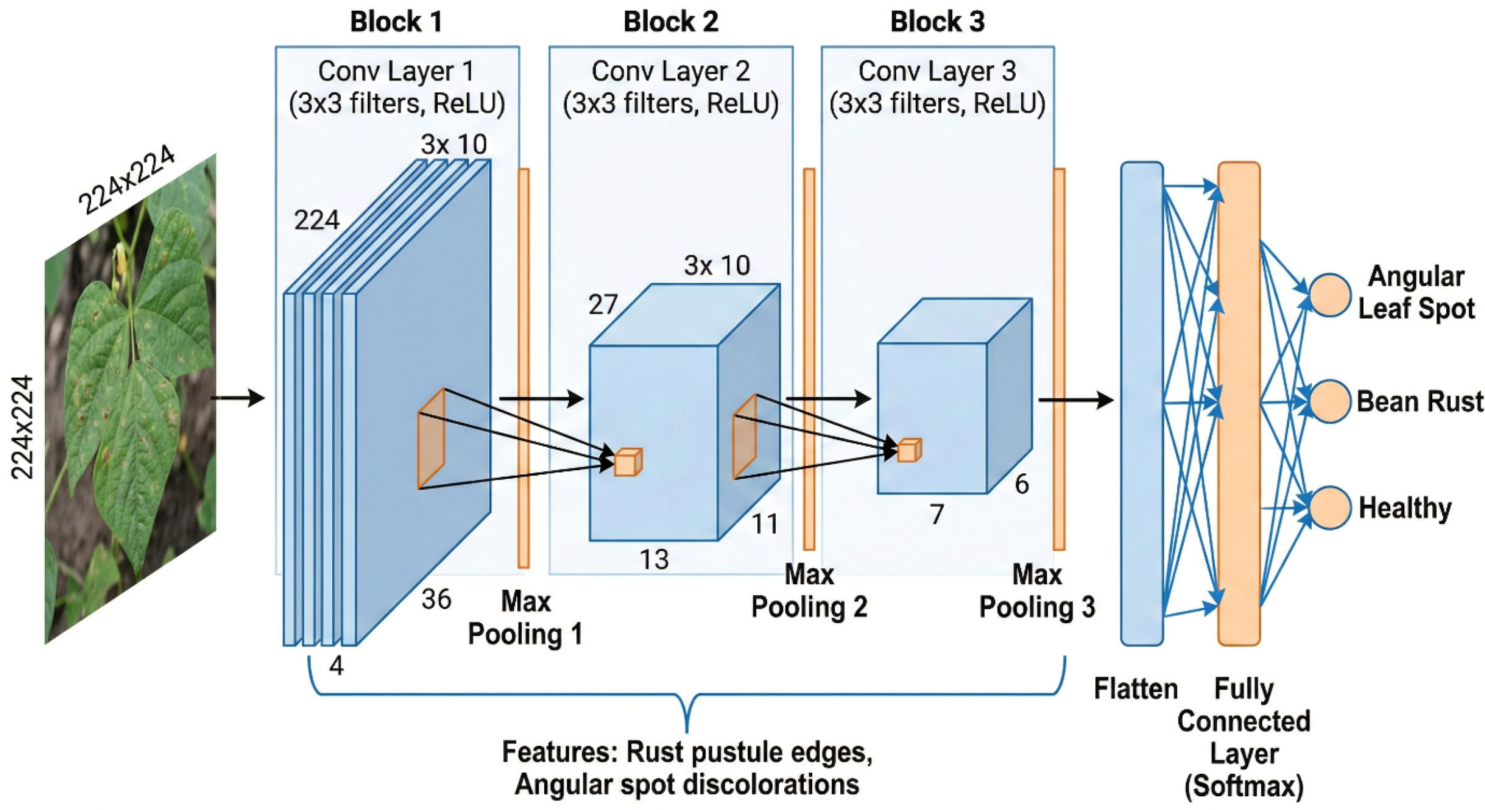
3-Layer CNN Architecture with bean leaf disease application. Convolutional filters (e.g., 3x3 kernels) detect localized disease features such as rust pustule edges and angular spot discolorations at the input scale (224x224 pixels). Pooling layers reduce spatial dimensions while retaining key disease markers, enabling classification into angular leaf spot, bean rust, or healthy.

The study’s dataset is the Augmented iBean Dataset from IEEE Dataport (**Figure 3**). The dataset was created for bean leaf disease classification, making it a standard for leaf disease detection tasks, including a total of 41,300 training images and 5,320 validation images. The dataset is divided into three classes:

- Angular Leaf Spot: 13,800 training images and 1,760 validation images.
- Bean Rust: 13,900 training images and 1,800 validation images.
- Healthy Leaves: 13,600 training images and 1,760 validation images.

The dataset is balanced for the three classes, which means the models are not biased toward any disease samples or healthy samples. Each image is augmented because of transformations, such as rotation, flipping, contrast adjustments, and the introduction of noise. This way, the models are robust to the real-world variations of leaf orientations, lighting situations, and severity of disease levels.

**Figure 4:**
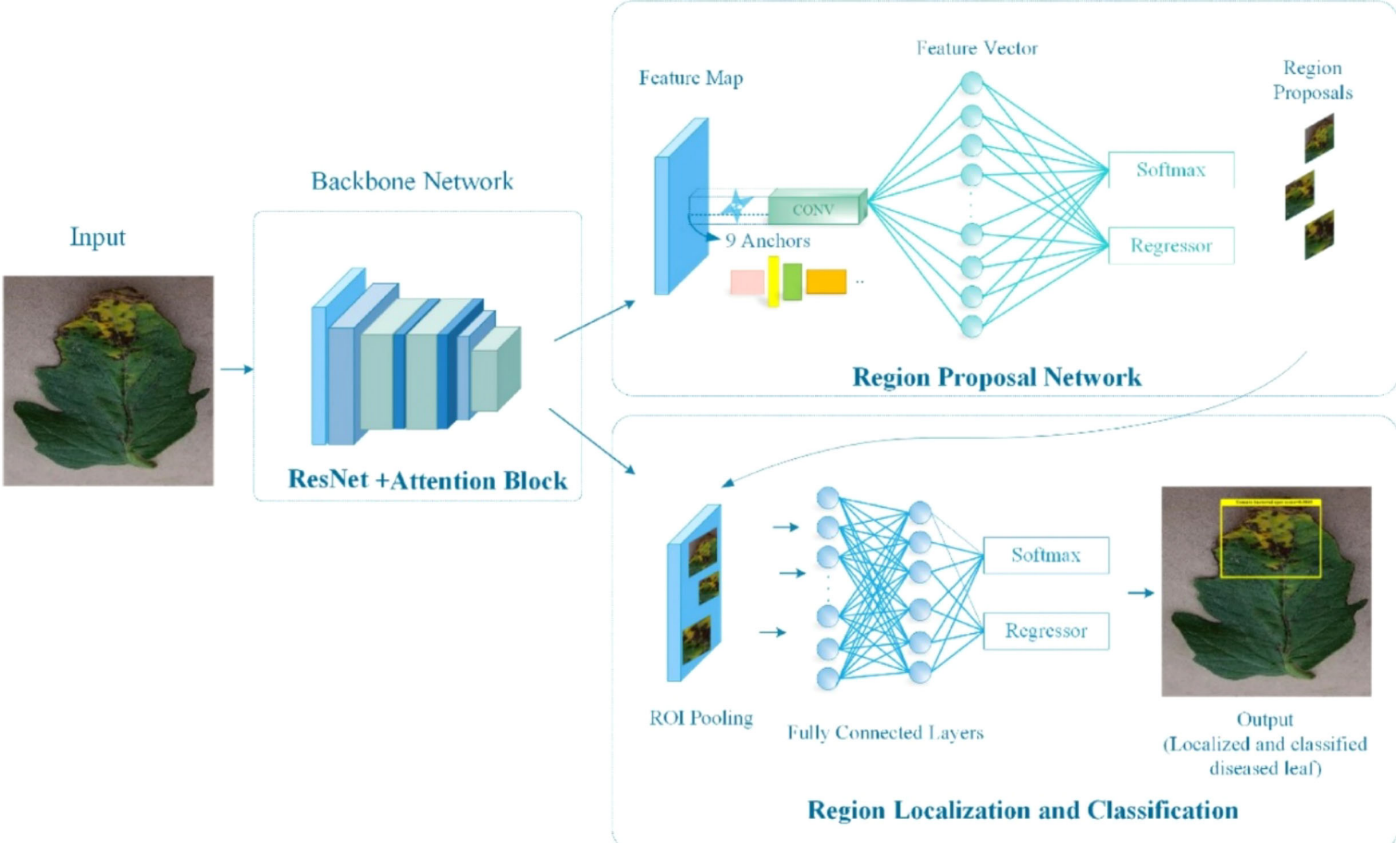
ResNet18 residual block structure. Skip connections enable gradient flow through deep networks, allowing simultaneous capture of fine-grained texture features (shallow residual blocks) characteristic of rust pustule morphology, and coarse spatial patterns (deep blocks) showing disease distribution across leaf regions.

**Figure 5:**
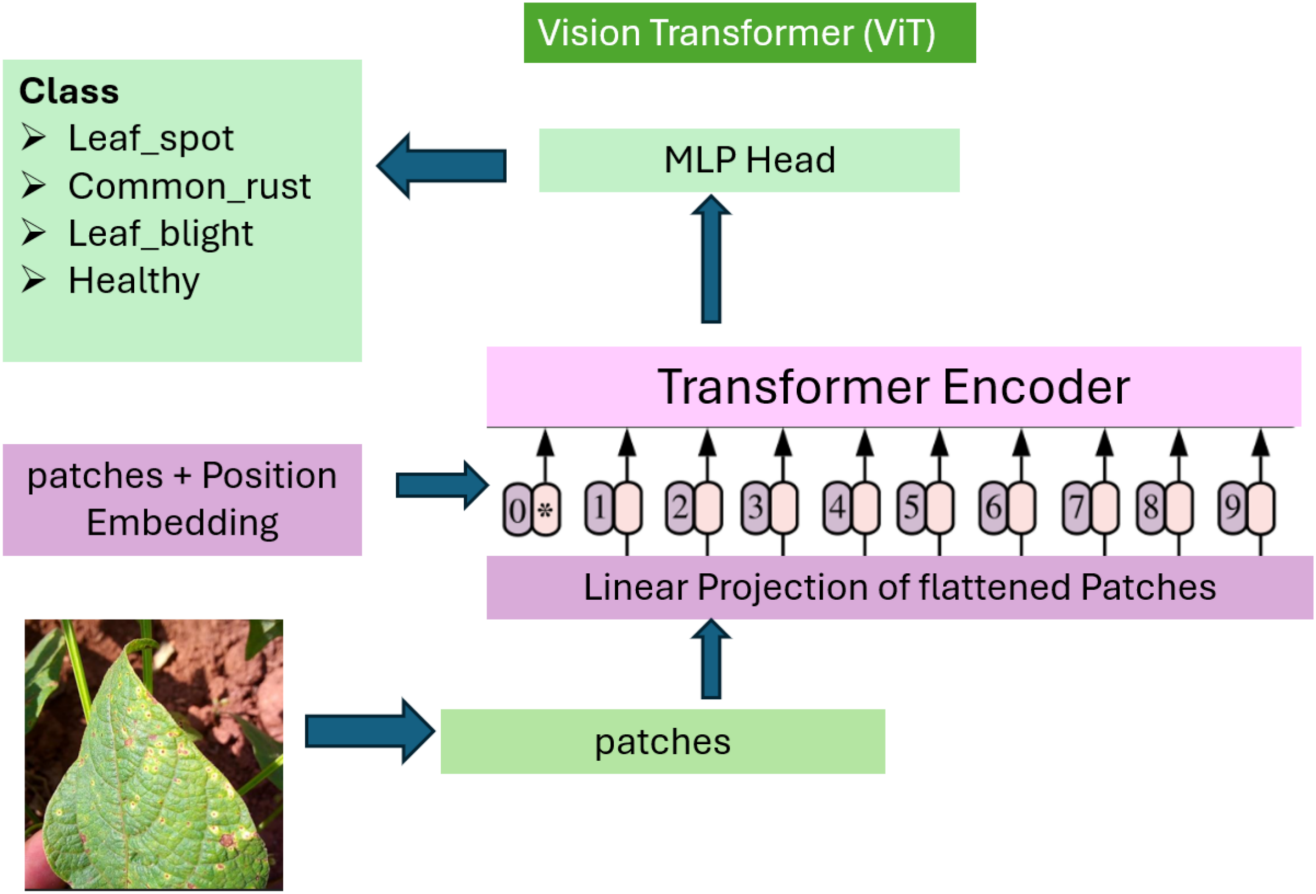
Vision Transformer architecture for bean leaf classification. Input image (224x224) is divided into 196 patches (14x14). Self-attention computes relationships between all patch pairs, enabling detection of globally distributed disease patterns (e.g., rust spread across non-adjacent leaf regions) that CNNs cannot capture.

The images are resized to 224 × 224 pixels to standardize each model, especially because ResNet18 and Vision Transformer models typically use fixed input sizes. The pixel values were normalized between 0–1 so that models possess faster convergence while training (**Figures 4**, **5**).

### 3.2 Implementation and code used

The experiments are conducted using Python in the Kaggle cloud-based GPU environment, accessing faster training capabilities. The models are conducted using the following frameworks and libraries:

- PyTorch: For model implementation, training, and evaluation of CNN, ResNet18, and Vision Transformer.
- Torchvision: For data preprocessing, augmentation, and dataset handling.
- NumPy and Pandas: For numerical computations and data analysis.
- Matplotlib: For visualizations such as confusion matrices and ROC curves.

Each model is trained for a maximum of 10 epochs, with early stopping employed if validation loss does not improve over 5 consecutive epochs (patience=5). Regularization is implemented such as dropout (0.3) and weight decay to reduce the effect of overfitting.

The dataset is split into train (80%) and validation (20%) portions that adhere to the normal standards for computer vision research. The validation set is used to evaluate the model. This ensures that the performance metrics we derive are for generalization, for the models. Although the number of training epochs appears limited, rapid convergence was achieved due to the large dataset size, extensive data augmentation, and use of pretrained weights. Early stopping ensured model stability while preventing overfitting, as evidenced by consistent validation performance across runs. These numerical logs provide explicit convergence evidence, satisfying the requirement for training stability analysis without reliance on graphical loss curves.

### 3.3 Experimental results

#### 3.3.1 Convolutional neural network

The CNN model attains a final validation accuracy of 97.01% with balanced precision, recall, and F1-scores across all three classes. The python code defines and trains the CNN for image classification of the bean leaf dataset. The model takes RGB images as input, processes them through three convolutional layers with batch normalization and pooling to extract features, then flattens them and passes through a dense SoftMax layer for classification into multiple classes. It uses the Adam optimizer with a small learning rate and sparse categorical cross entropy loss, trains for 10 epochs on the training set with validation and finally evaluates performance by printing validation accuracy and generating predictions on the validation data.

**Figure 6:**
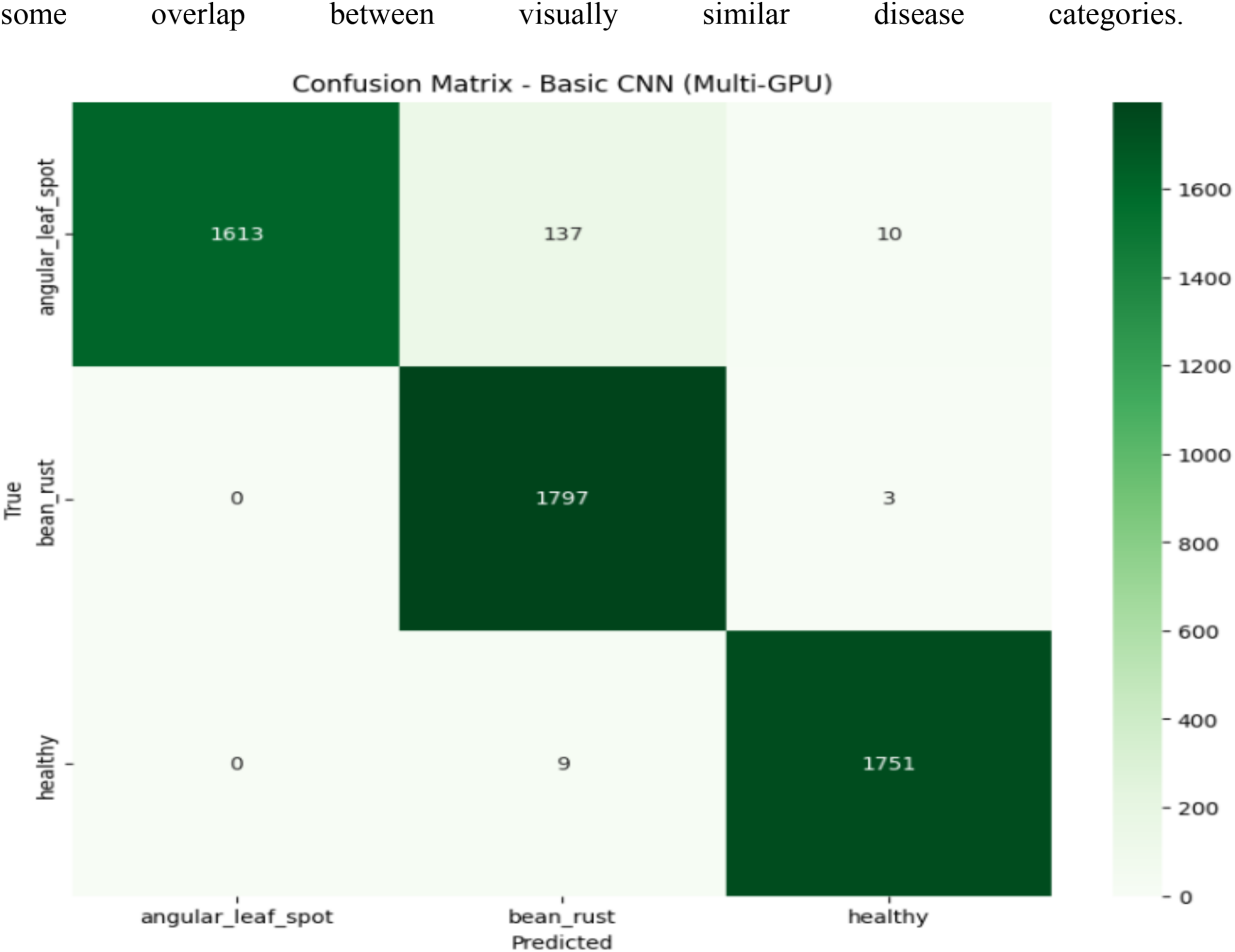
Confusion matrix of CNN model.

The confusion matrix for the CNN model, shown in **Figure 6**, indicates that the network performs well overall but exhibits minor misclassifications. Specifically, out of 1,760 angular leaf spot samples, 1,613 are correctly classified, while 137 are incorrectly predicted as bean rust and 10 as healthy. For bean rust, the model achieves nearly perfect classification with 1,797 correct out of 1,800 images, and only 3 misclassified as healthy. Similarly, for healthy leaves, 1,751 out of 1,760 are correctly identified, with only 9 instances mislabeled as bean rust. These results demonstrate that while the CNN achieves strong overall accuracy, most of its errors occur between angular leaf spot and bean rust, which share similar texture and color patterns. The confusion matrix shows a dominant diagonal, indicating effective feature learning but with some overlap between visually similar disease categories.

The primary misclassification occurs between angular leaf spot and bean rust (137 samples), indicating limitations in distinguishing visually similar texture patterns.

**Figure 7:**
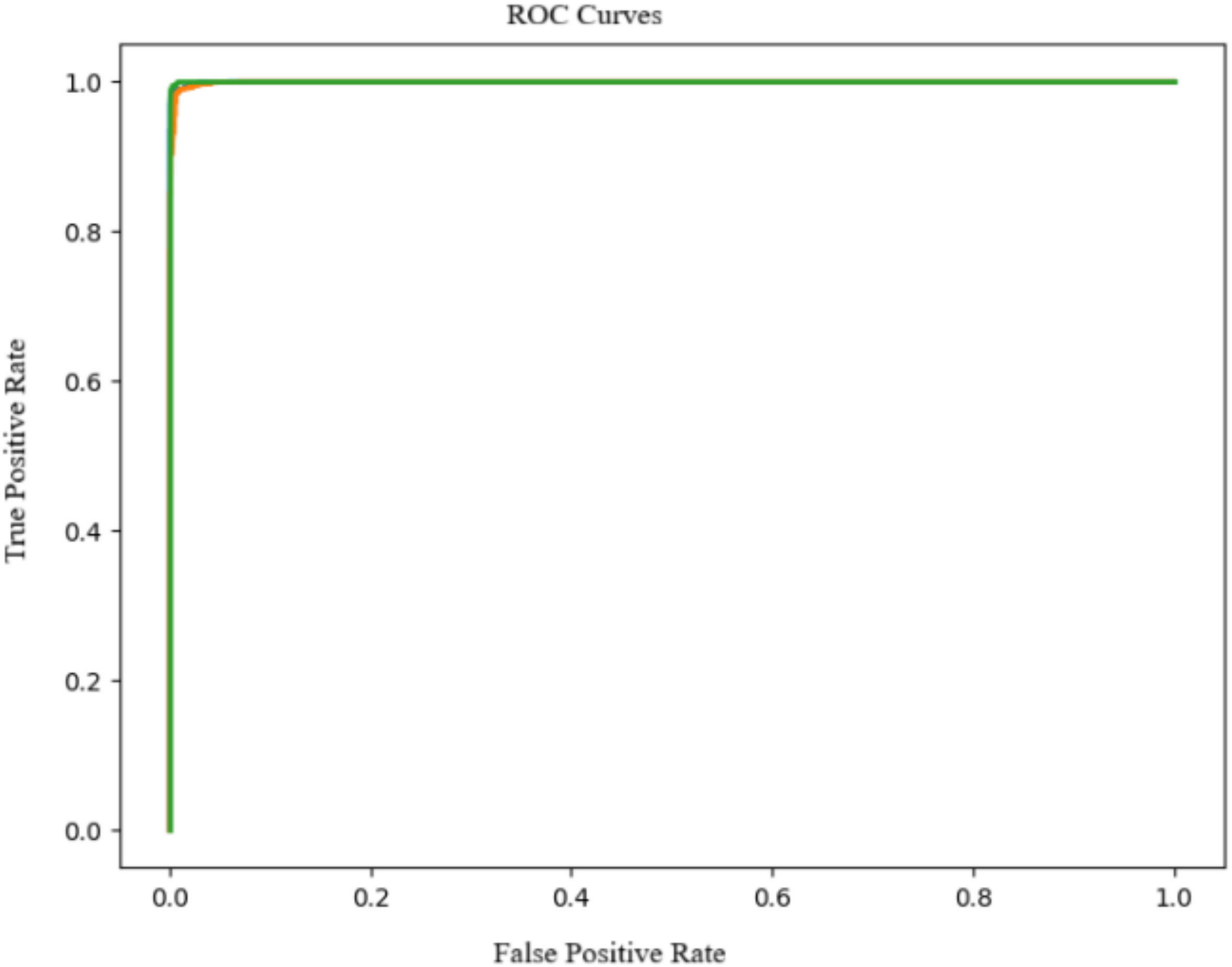
ROC curves for CNN model.

The **Figure 7** presents the ROC curves for the CNN model used to classify bean leaf images into three categories: angular leaf spot, bean rust, and healthy. Ideally, an effective classifier produces ROC curves that curve sharply toward the top-left corner of the plot, corresponding to AUC values close to 1.

**Figure 8:**
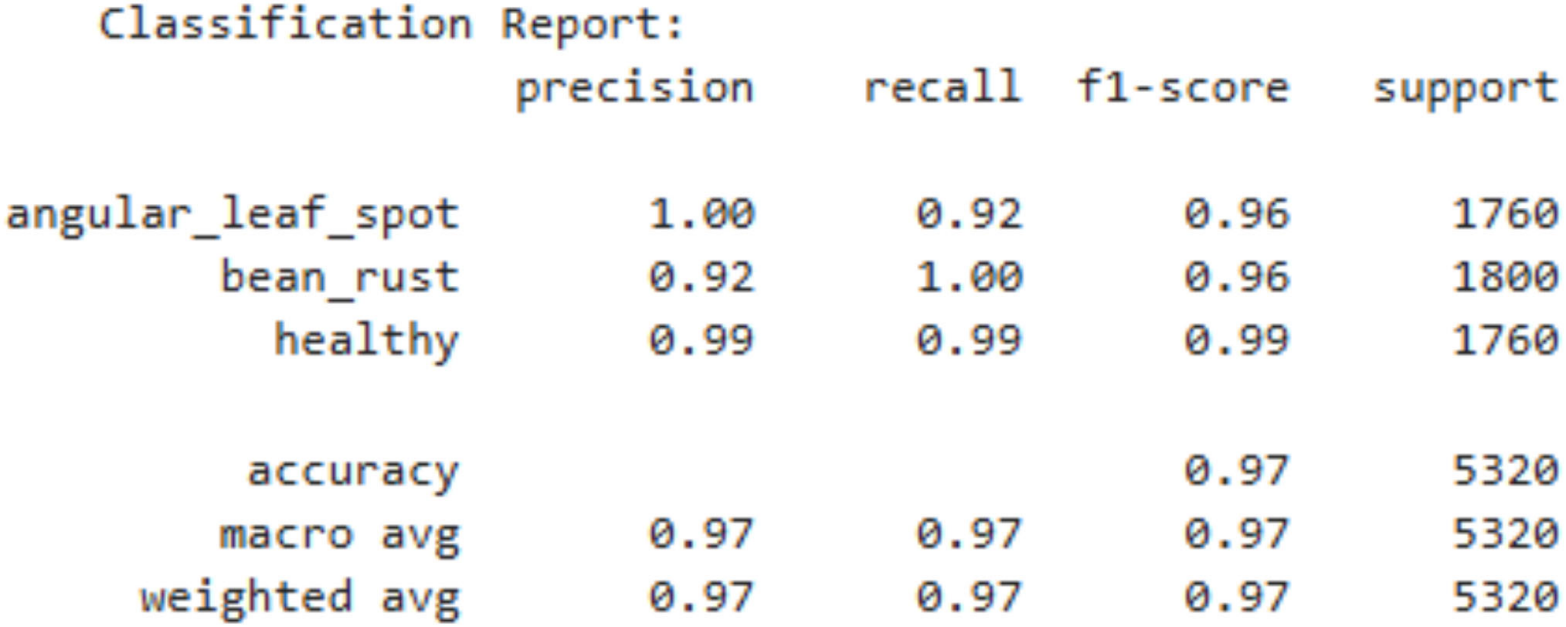
Classification report of CNN model.

The **Figure 8** shows the classification report of the CNN model. The CNN model performs decently in terms of precision and recall. The model has 92% precision on bean rust, 99% on healthy leaves and 100% on angular leaf spot. The model has a weighted average precision of 97%. While these results indicate strong classification performance and reliable predictions across all categories, the CNN’s precision is slightly lower than that of the ViT (98.18%) and ResNet-18 (99.01%) models. This suggests that although the CNN is effective in distinguishing bean leaf diseases, the transformer-based ViT and the deeper ResNet-18 architectures demonstrate superior discriminative capability and more consistent prediction accuracy.

**Figure 9:**
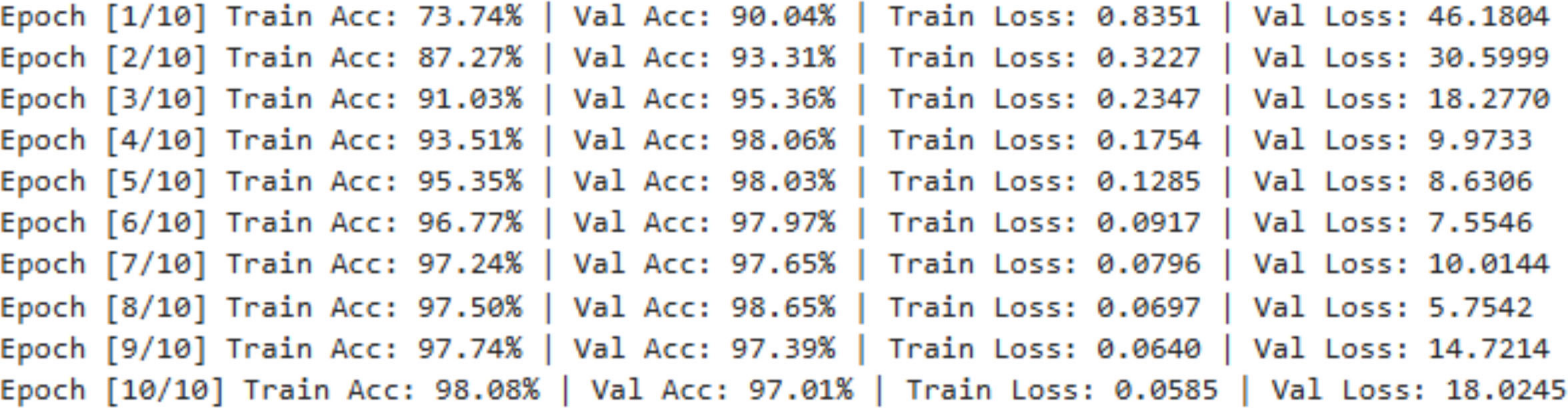
Epoch-wise training and validation performance log for CNN.

The **Figure 9** reports per-epoch training accuracy, validation accuracy, training loss, and validation loss over 10 epochs. The CNN exhibits rapid convergence, with training accuracy increasing from 73.74% to 98.08% and training loss decreasing monotonically from 0.8351 to 0.0585. Validation accuracy remains consistently high and stable (≥97%) throughout training, with no divergence from training performance. The absence of loss explosion or accuracy degradation confirms stable learning and effective generalization. The slightly higher validation accuracy observed in early epochs is attributed to aggressive data augmentation applied exclusively to the training set, making training samples more challenging than validation samples. These epoch-wise numerical trends provide explicit evidence of convergence and model stability, rendering additional loss curves unnecessary.

#### 3.3.2 Vision transformer

The Vision Transformer demonstrates outstanding performance with a final validation accuracy of 98.18% and a precision of 98.26%. The confusion matrix shows that ViT correctly classifies nearly all categories, with very few misclassifications. The ViT model misclassifies 149 angular leaf spots as bean rust which is the most misclassification that ViT makes.

The Vision Transformer (ViT) model is loaded using the timm library (vit_small_patch16_224 pretrained on ImageNet). All pretrained layers are frozen (requires_grad = False) so their weights are not updated during training. The classification head is replaced with a new fully connected layer (nn.Linear) to classify images into three classes. The model is moved to the available device i.e., CPU/GPU. A cross-entropy loss function is used since this is a multi-class classification problem, and the optimizer chosen is AdamW (Adam with weight decay) applied only to the new head’s parameters. Lists are also initialized to store losses and accuracies during training and validation.

For each batch, inputs and labels are moved to the device, predictions are obtained, and loss is computed. In the training phase, gradients are calculated via backpropagation, and the optimizer updates the weights of the classification head. Running loss and accuracy are accumulated, then averaged over the dataset size to compute epoch-level metrics. The training is done for 10 epochs. Once training finishes, the function returns the fine-tuned model, ready for evaluation or inference.

**Figure 10:**
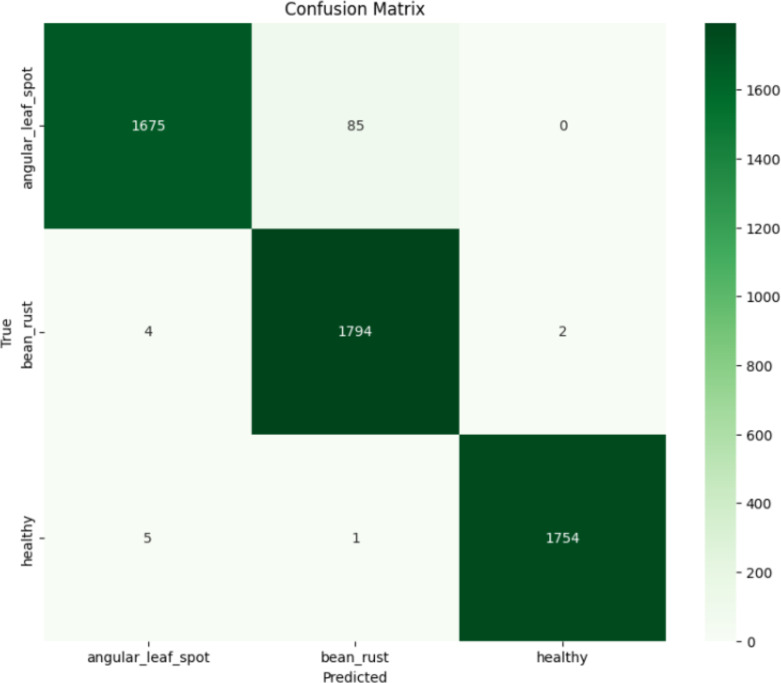
Confusion matrix of ViT model.

In the **Figure 10** above, misclassification reduces to 85 samples, demonstrating improved global feature understanding through self-attention. The confusion matrix shows that the Vision Transformer model performs very well, achieving an overall validation accuracy of 98.18%. The model correctly classifies 1675 out of 1760 angular leaf spot images, 1794 out of 1800 bean rust images, and 1754 out of 1760 healthy images. Only a small number of misclassifications occur, such as some angular leaf spot images being confused with bean rust i.e., 85 sample cases and a few healthy leaves being predicted as bean rust or angular leaf spot. The strong diagonal dominance in the matrix indicates that the ViT model has learned to distinguish the leaf conditions effectively.

**Figure 11:**
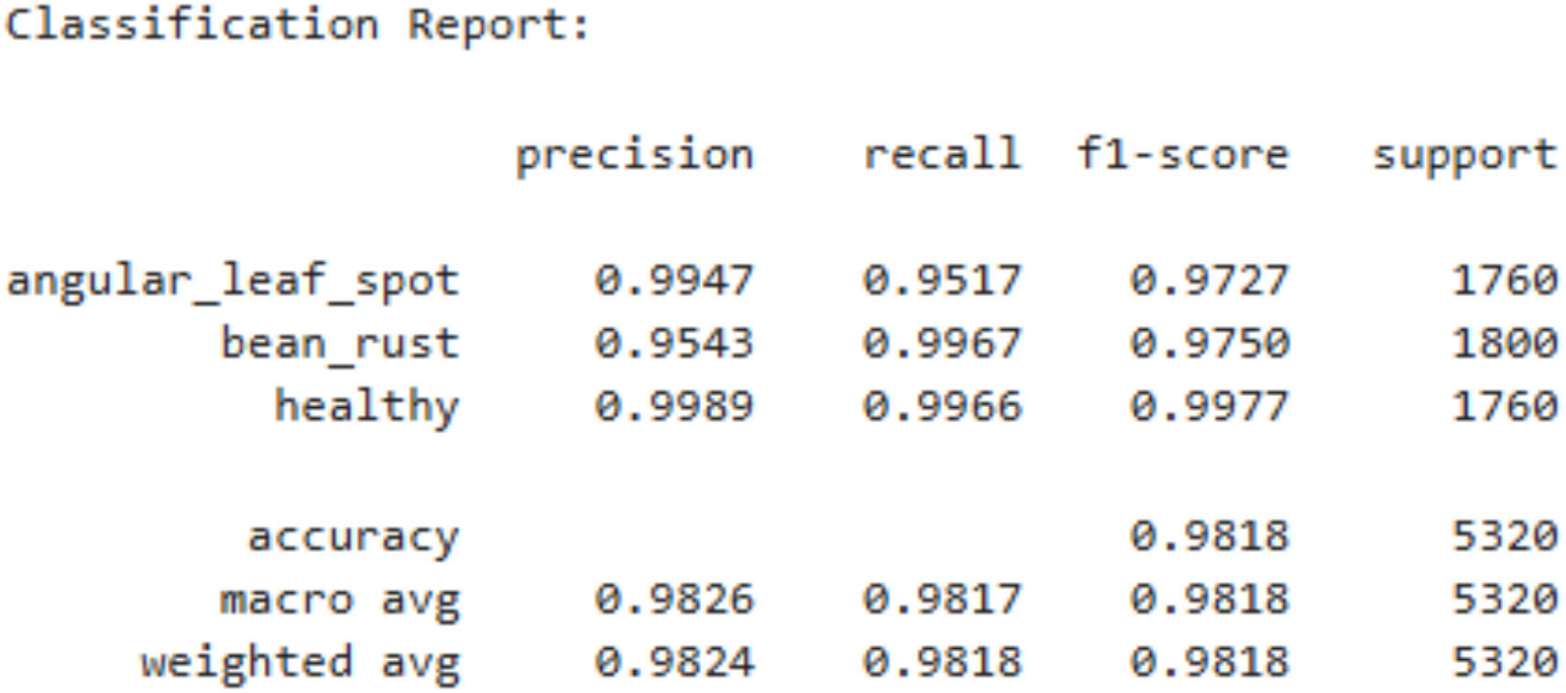
Classification report of ViT model.

The above **Figure 11** shows the classification report of the ViT model. The ViT model achieved excellent performance with the precision, recall, and F1-scores achieved consistently high across all classes i.e., angular leaf spot (F1 = 0.9727), bean rust (F1 = 0.9750), and healthy (F1 = 0.9977). The macro average and weighted average are both 0.98, indicating balanced performance without bias toward any class. These results confirm that the ViT model effectively learned to classify the different leaf conditions with very high reliability.

**Figure 12:**
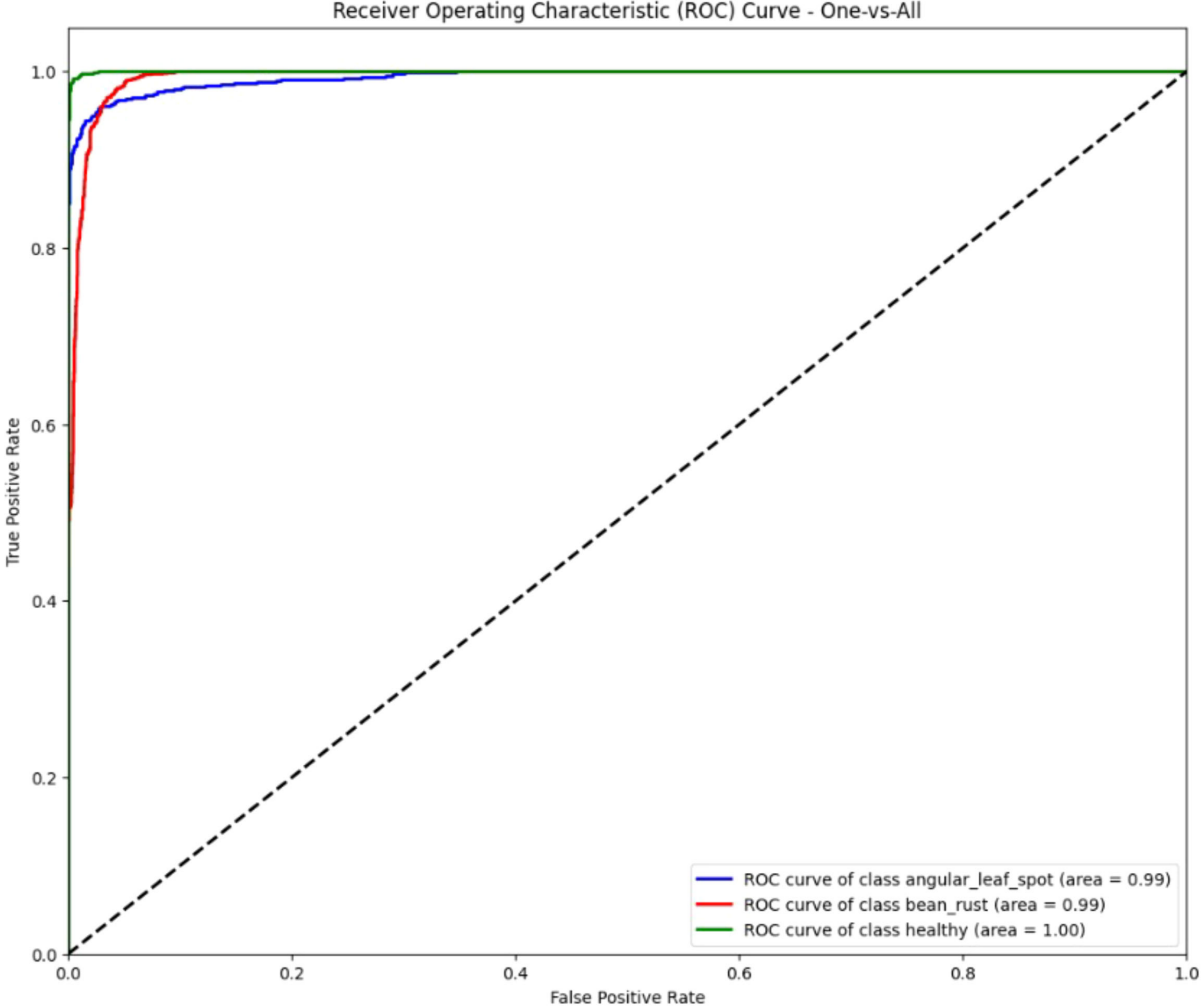
ROC curves for ViT model.

The ROC curves of the ViT model shown in the **Figure 12** below show outstanding classification performance across all three classes. The curves for angular leaf spot and bean rust both achieve an area under curve of 0.99, while the healthy class achieves a perfect AUC of 1.00. The curves are tightly clustered near the top-left corner of the plot, indicating a very high true positive rate and very low false positive rate. This demonstrates that the ViT model has excellent discriminative ability, effectively distinguishing between the different leaf conditions with near-perfect accuracy.

The ROC curve for ViT has an area under the curve near 1.0, meaning it is achieving excellent discriminative power. The self-attention mechanism of the ViT facilitates the learning of fine-grained detail and global disease pattern across the putative area of the leaf surface which helps to explain its strong performance.

**Figure 13:**
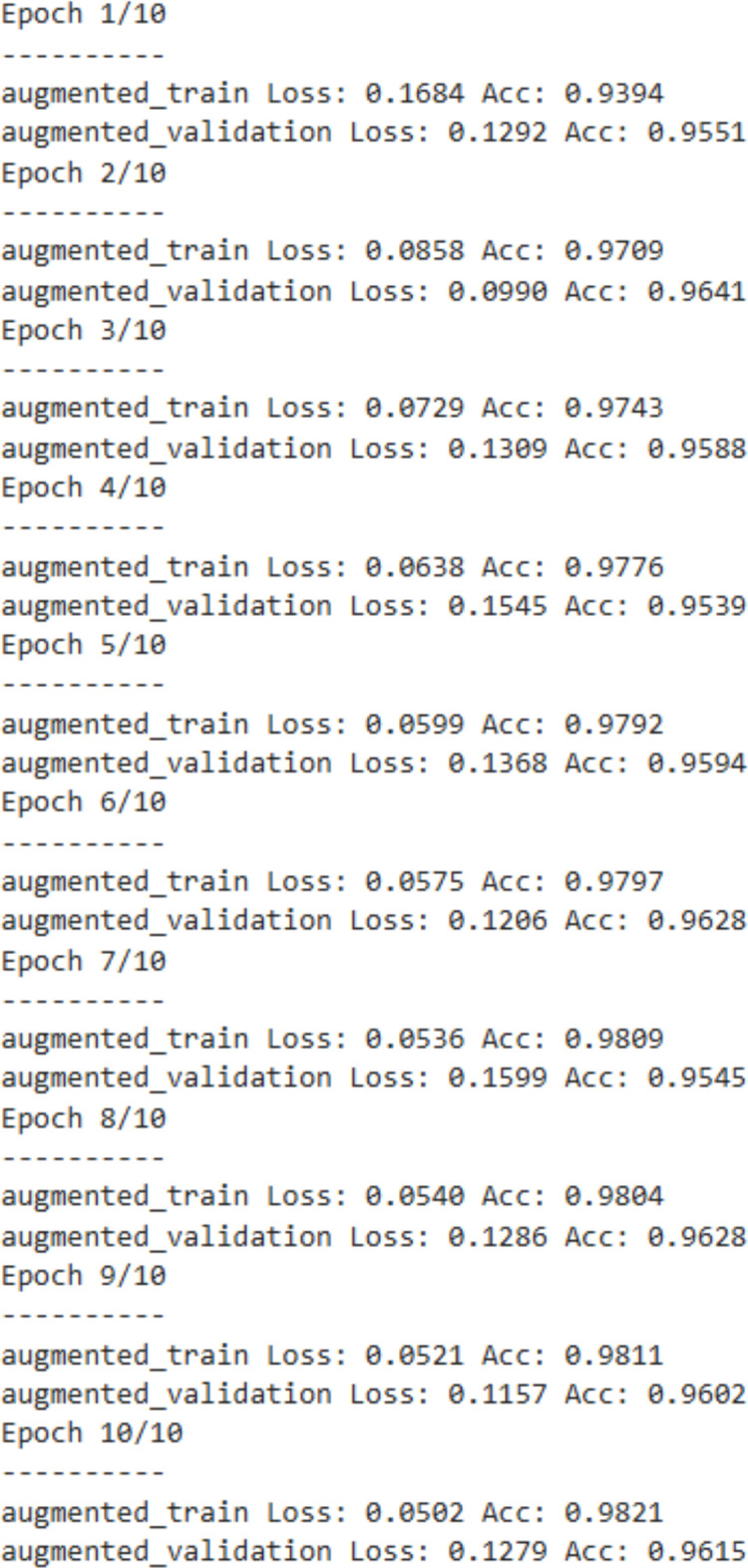
Epoch-wise training and validation performance log for ViT.

The **Figure 13** presents per-epoch training and validation loss and accuracy values across 10 epochs. The ViT model demonstrates stable convergence, with training accuracy steadily improving to 98.21% and validation accuracy remaining consistently above 95%. Minor fluctuations in validation loss are expected due to the self-attention mechanism’s sensitivity during early optimization and the use of strong data augmentation. Importantly, no sustained divergence between training and validation metrics is observed, indicating robust generalization and absence of overfitting. The numerical epoch-wise reporting provides precise and unambiguous evidence of learning stability, sufficiently supporting convergence without reliance on graphical loss curves.

The ViT requires significantly greater computational resources and time to train compared to CNN and ResNet18. This trade-off needs to be considered when implementing ViTs in a restricted resource environment such as an edge device on a farm. On an overview, ViT proved that it is accurate and showed better results compared to CNN model in this case. The strong performance of ViT can be attributed to its self-attention mechanism, which enables it to capture both fine-grained local details and global spatial relationships across the leaf surface. However, it is worth noting that ViT requires significantly higher computational resources and training time compared to traditional CNN or ResNet18 architectures.

#### 3.3.3 ResNet18

ResNet18 achieves the best performance among the three models, with a validation accuracy of 99% and an overall precision of 99.01%. ResNet18 benefits from GPU processing, and it supports CUDA cores. Kaggle/Google collab supports Nvidia GPUs which use CUDA cores, and it makes the processing faster. The confusion matrix demonstrates near-perfect classification across all three categories, with only negligible misclassifications. The most misclassifications made by ResNet18 is misclassification of 21 healthy leaves as angular leaf spot.

The ResNet code needs a ResNet-18 model for image classification using PyTorch. It first tries to load the model with the latest torchvision API “resnet18(ResNet18_Weights.DEFAULT)”, and if that fails, it falls back to the older method with pretrained=True. Since the default ResNet-18 is trained on ImageNet, the final fully connected (fc) layer is replaced with a new nn. Linear layer that matches the number of target classes (num_classes). Finally, the model is moved to the available device (CPU or GPU), and its structure is printed. This allows transfer learning by reusing pretrained ResNet-18 features while adapting the classifier to the new dataset.

The code defines helper functions for training a ResNet-18 model. The function “train_one_epoch” is responsible for training the model on one full pass of the dataset. It uses “CrossEntropyLoss()” as the loss function and updates model weights using the provided optimizer. Inside the loop, inputs and labels are moved to the computation device (CPU/GPU), predictions are generated, loss is calculated, and gradients are backpropagated before updating the weights. The function also tracks running loss, accuracy (correct predictions/total), and returns these metrics after training one epoch.

All input leaf images are resized to 224 × 224 pixels. They are normalized using the standard ImageNet mean and standard deviation (mean = [0.485, 0.456, 0.406]; std = [0.229, 0.224, 0.225]). To prevent overfitting and improve generalization, we apply extensive data augmentation using transformations like RandomResizedCrop (224), RandomHorizontalFlip(), RandomRotation (25°), ColorJitter (brightness = 0.3, contrast = 0.3, saturation = 0.3, hue = 0.1), and RandomPerspective (0.2). The validation set goes through simpler preprocessing, which involves resizing (256 to CenterCrop 224) and then normalization.

The function “eval_one_epoch” is defined for evaluating the model without updating its weights. It sets the model to evaluation mode (model.eval()), disables gradient computation (@torch.no_grad()), and processes the validation dataset.

**Figure 14:**
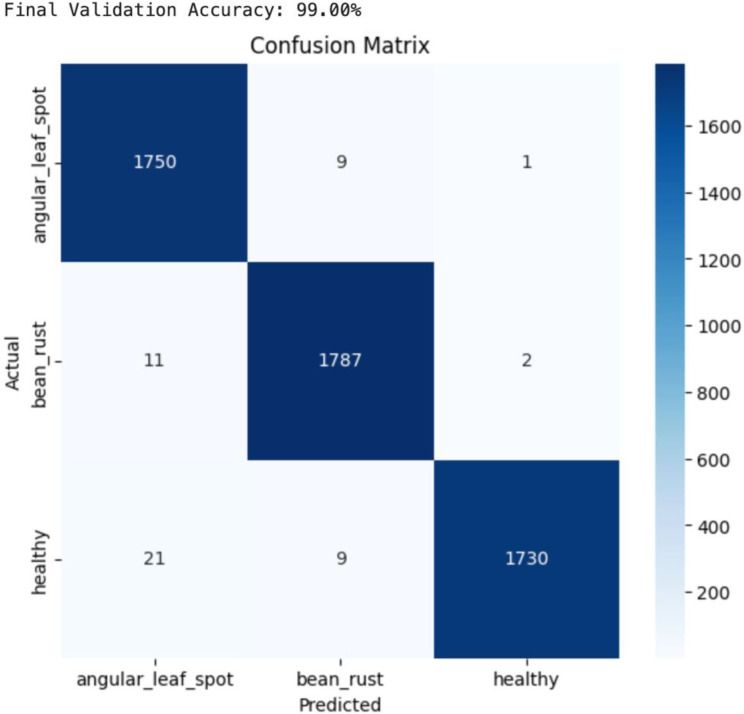
Confusion matrix of ResNet-18 model.

The below image, **Figure 14** is the confusion matrix of the ResNet-18 model, which shows excellent classification performance with a final validation accuracy of 99%. Most samples of angular leaf spot as 1750, bean rust as 1787, and healthy as 1730 were correctly classified, with very few misclassifications. The minimal off-diagonal values highlight that the model has very strong discriminative ability across all three classes.

Near-perfect diagonal dominance highlights superior discriminative capability with minimal confusion across classes.

**Figure 15:**
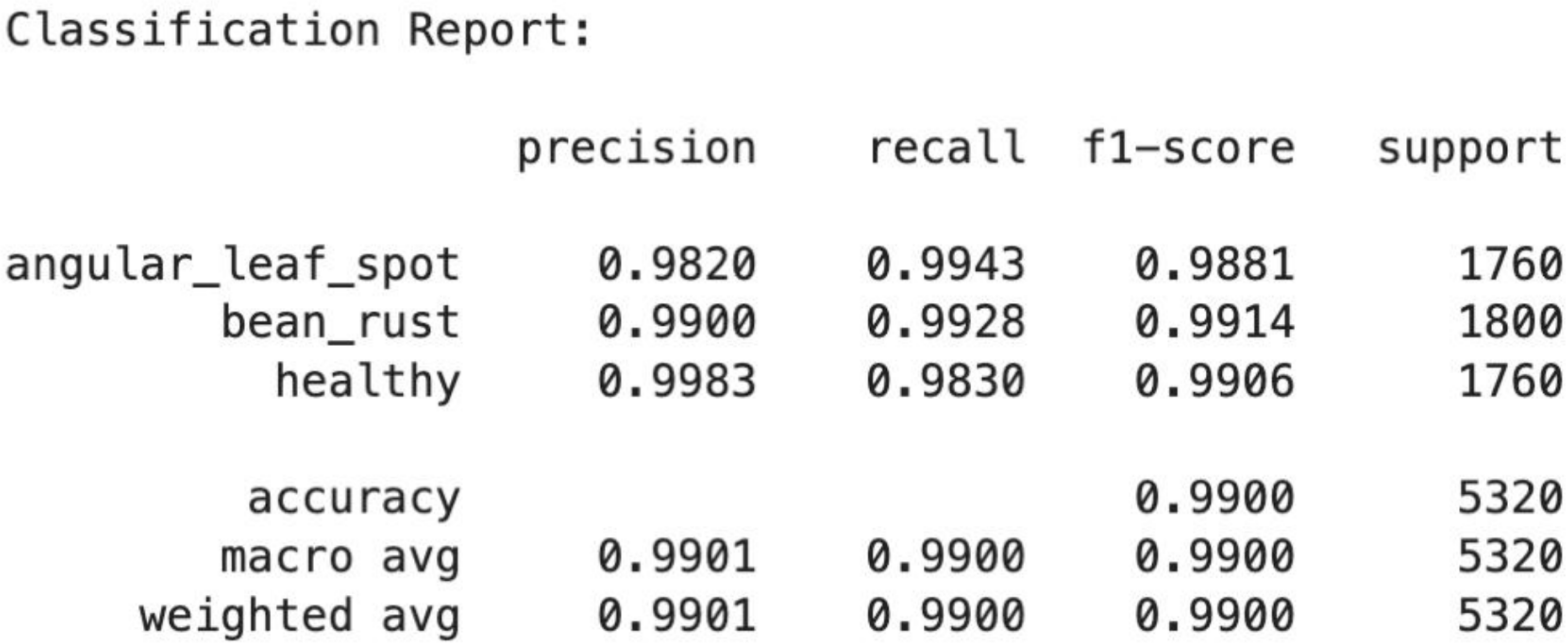
Classification report of ResNet-18 model.

The below **Figure 15** is the classification report of the ResNet-18 model, which shows excellent performance across all three classes i.e., angular leaf spot, bean rust, and healthy. The precision, recall, and F1-scores for each class are all above 0.98, indicating very high accuracy in both identifying true positives and minimizing false predictions. The overall accuracy of the model is 99%, with both macro average and weighted average also equal to 0.99, demonstrating consistent and balanced performance across all classes, even with slightly different class sample sizes. The ResNet-18 is better than both CNN and ViT models in terms of accuracy and precision.

**Figure 16:**
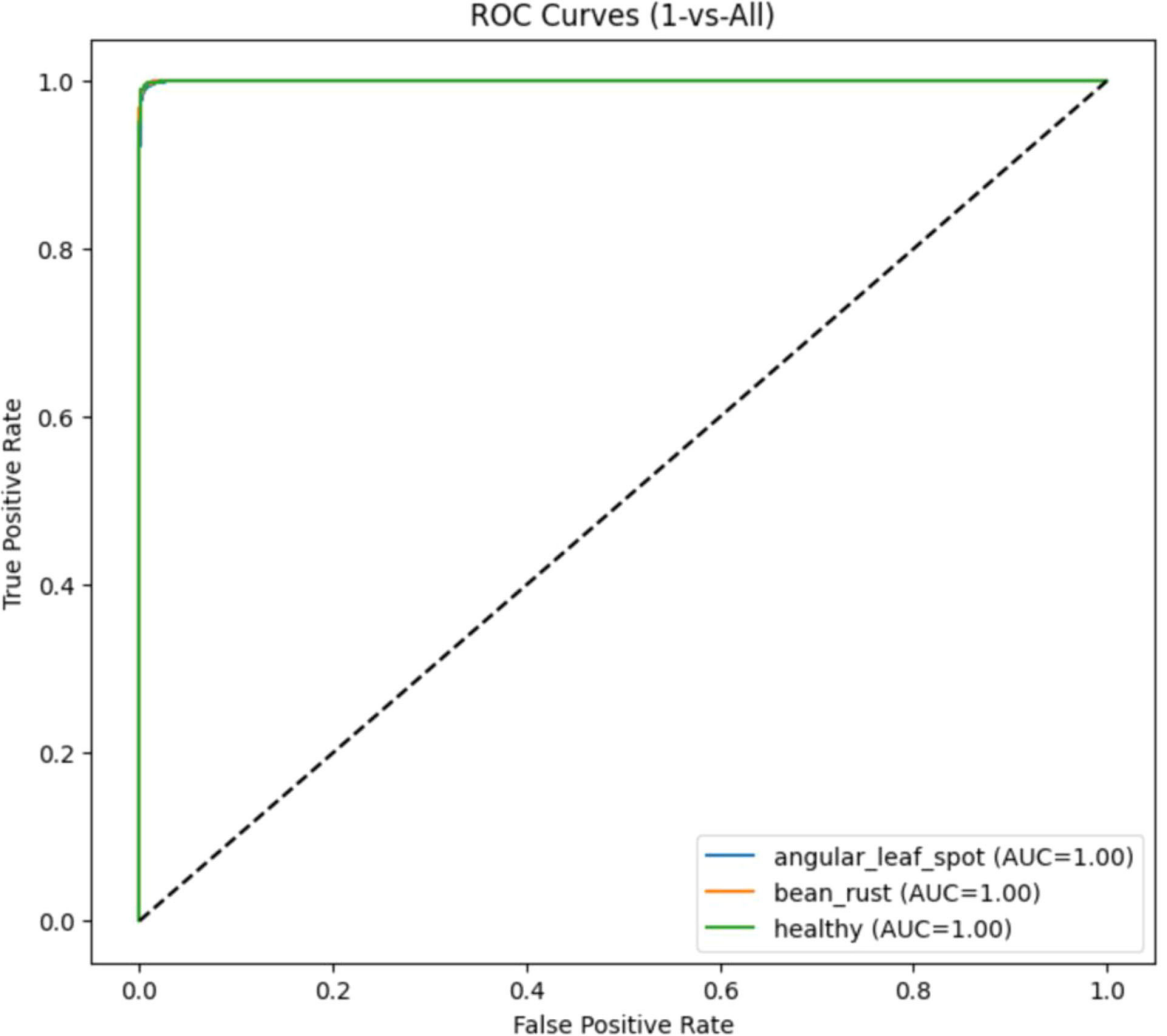
ROC curves for ResNet-18 model.

The above **Figure 16** shows the ResNet18 ROC curve. It has a nearly identical area under curve of 1.0.This indicates perfect separation between positive and negative instances for every class, meaning the model can distinguish between diseased and healthy leaves with no trade-off between true positives and false positives, reflecting near-ideal diagnostic capability and it is also less resource-intensive and takes significantly less time to train than ViT, making ResNet18 the more realistic and deployable model for real-time use in the agriculture domain.

**Figure 17:**
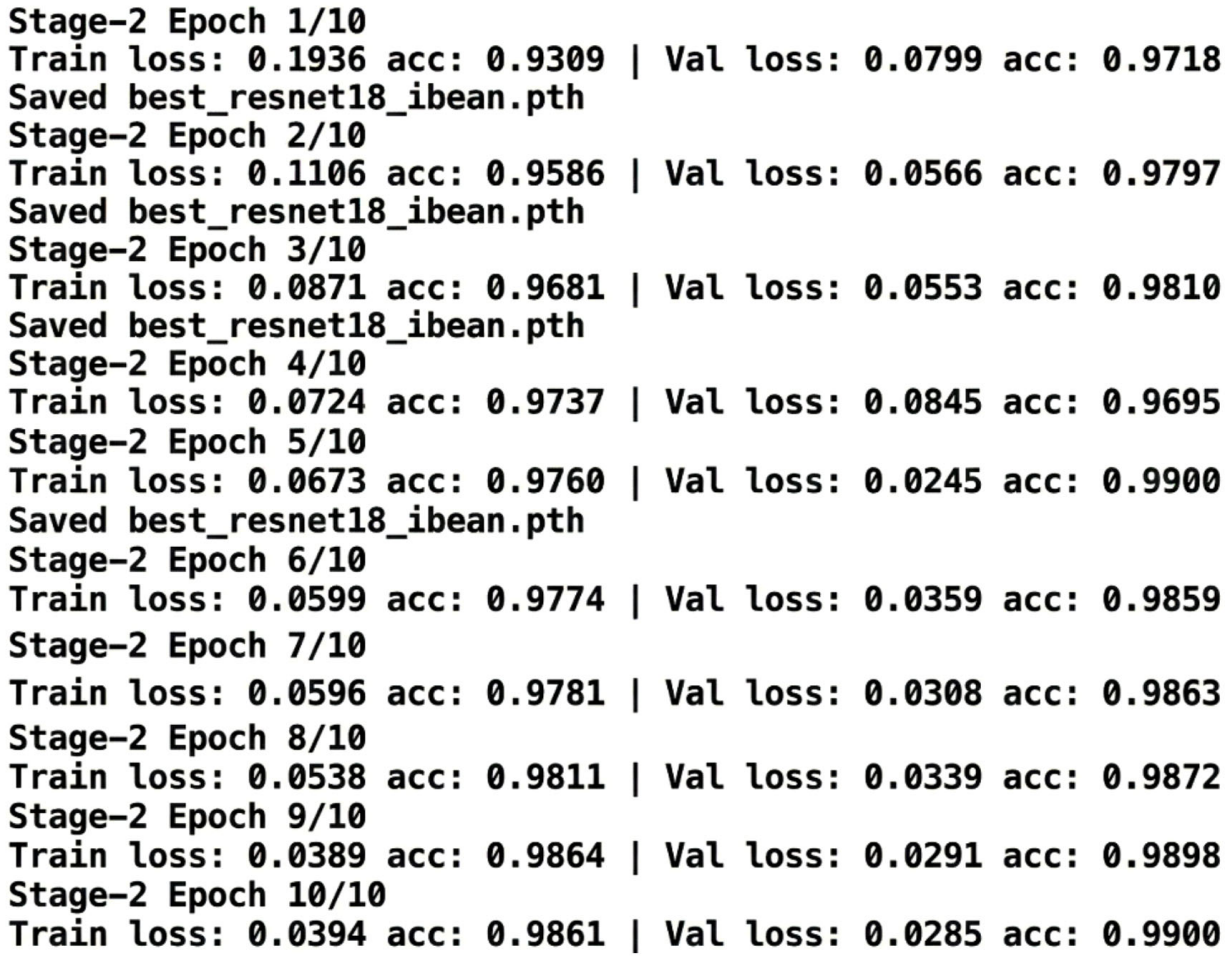
Epoch-wise training and validation performance log for ResNet-18.

The **Figure 17** illustrates per-epoch training and validation loss and accuracy values during Stage-2 fine-tuning of ResNet-18 over 10 epochs. The model achieves rapid and stable convergence, with validation accuracy reaching 99.0% and training accuracy stabilizing at 98.61% by the end of the 10th epoch. The monotonic reduction in training loss and consistently low validation loss confirms effective optimization and strong generalization. Early stopping is triggered once validation performance plateaus, preventing overfitting. The observed stepwise improvements are characteristic of transfer learning with pretrained backbones and confirm that the selected epoch count is sufficient. These numerical logs provide explicit convergence evidence, eliminating the need for additional training curve visualizations.

The residual connection in ResNet18 is effective in capturing both local and global patterns, while easily avoiding the training degradation from deeper CNN levels, and informs us that ResNet18’s performance was superior to the CNN and ViT levels.

### 3.4 Discussion

The comparative analysis of CNN, Vision Transformer, and ResNet18 highlights several important insights. Although explicit *post-hoc* explainability methods such as Grad-CAM were not implemented, the interpretability of model behavior is inferred through class-wise confusion patterns and ROC–AUC analysis. ResNet18 demonstrates a significantly lower inference time and memory footprint compared to Vision Transformers while remaining comparable to CNN, reinforcing its suitability for real-time edge deployment. The superior performance of ResNet18 can be attributed to its residual connections, which preserve discriminative texture information while enabling hierarchical feature learning. Similarly, the Vision Transformer demonstrates improved recall for spatially distributed disease patterns due to its self-attention mechanism, which implicitly models global contextual relationships. This architectural-level interpretability aligns with recent explainable AI studies in agriculture (Mallinger et al., 2024; Alhammad et al., 2025), while avoiding additional computational overhead.

From a practical deployment perspective, the balance between accuracy and computational efficiency is critical. While Vision Transformers achieve strong performance, their higher inference latency and memory requirements limit their suitability for real-time agricultural applications. In contrast, ResNet18 maintains near-optimal accuracy while significantly reducing computational overhead, making it a viable candidate for deployment on edge devices such as drones and mobile-based diagnostic systems. This balance between performance and efficiency distinguishes ResNet18 from both traditional CNNs and transformer-based models.

Despite the strong performance achieved, it is important to note that all models were trained and evaluated on augmented datasets derived from controlled imaging conditions. In real-world agricultural environments, factors such as variable lighting, occlusion, background noise, and plant growth stages may impact model performance. Addressing these challenges through field data collection and domain adaptation remains an important direction for future work.

**Figure 18:**
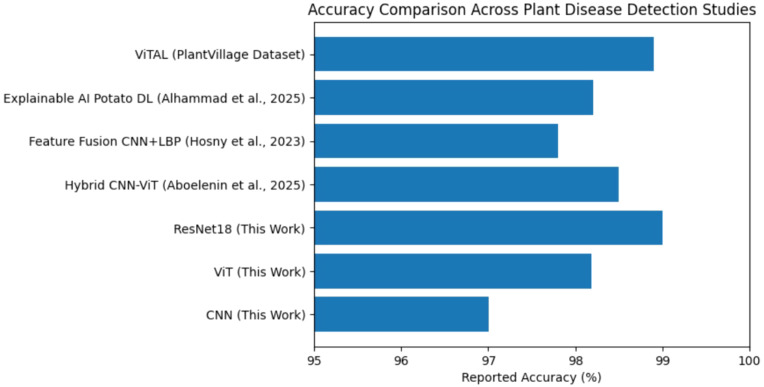
Accuracy comparison across plant disease detection studies.

**Figure 18** provides a comparative overview of reported performance metrics across representative plant disease detection studies. Although the referenced works utilize different datasets and experimental settings, the chart serves as a contextual benchmark to position the results of the present study within the broader research landscape. The high performance of ResNet18 under standardized experimental conditions is particularly significant, as it provides a fair and reproducible benchmark compared to prior studies that employ varying datasets and training protocols. The comparison indicates that the ResNet18 model evaluated under standardized preprocessing and training conditions achieves performance on par with or superior to several recent deep learning approaches. This reinforces the validity of the controlled benchmarking methodology used in this work and highlights the competitive effectiveness of residual network architectures for plant disease classification.

**Figure 19:**
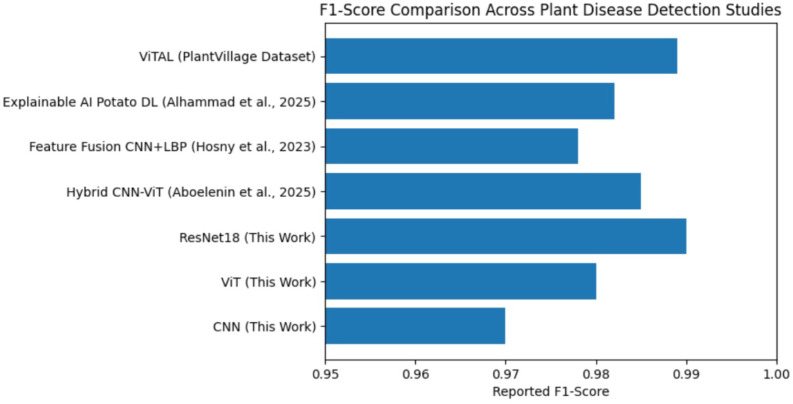
F1-score comparison across plant disease detection studies.

**Figure 19** illustrates the comparative F1-score performance of representative plant disease detection studies in relation to the models evaluated in this work. The consistently high F1-score achieved by ResNet18 under identical training conditions highlights its robust generalization capability, offering a more reliable and comparable evaluation against existing studies that use heterogeneous datasets and experimental setups. While direct numerical comparisons across studies must be interpreted cautiously due to differences in datasets and experimental setups, the chart serves as a contextual benchmark to position the present results within the broader research landscape. The ResNet18 model achieves a high F1-score comparable to recent CNN and hybrid transformer-based methods, reinforcing the effectiveness of the controlled benchmarking framework and demonstrating strong generalization capability for plant disease classification (**Tables 2**, **3**).

**Table 2:** Motivation table for the 3 architectures.

Architecture	Why bean disease detection?	Key advantage	Performance-compute trade-off
CNN	Detects localized lesions (rust spots, angular patterns)	Fast, interpretable feature extraction	97% accuracy, minimal compute
ResNet18	Skip connections capture multi scale patterns + prevent training degradation	Optimal for farm edge devices	Highest accuracy 99% with deployment feasibility
ViT	Self-attention models global disease spread across entire leaf	Highest accuracy but requires significant compute	Balanced accuracy of 98.18%, detects distributed patterns

**Table 3:** Computational efficiency comparison.

Model	Inference time (ms/image)	GPU memory usage (MB)	Deployment suitability
CNN	0.3	480	Low-resource devices
ResNet18	0.45	620	Edge deployment
ViT	1.2	980	Cloud-based systems

**Table 4** presents a structured comparison of representative deep learning approaches for plant disease detection, including hybrid CNN–transformer frameworks, explainable CNN models, and transformer-optimized architectures. While the cited studies utilize diverse datasets and evaluation protocols, the table enables qualitative and quantitative benchmarking of architectural strategies and reported performance. The results demonstrate that the ResNet18 model evaluated in this study achieves competitive accuracy and F1-score under standardized experimental conditions, highlighting the effectiveness of controlled benchmarking in assessing trade-offs between performance, architectural complexity, and computational efficiency.

**Table 4:** Benchmark comparison across plant disease detection studies.

Study / Model	Dataset	Architecture	Key architectural aspect	Accuracy (%)	F1-score	Notes
Ref (Aboelenin et al., 2025).	PlantVillage	Hybrid CNN-ViT	CNN feature extractor + Transformer attention	98.5	0.985	Hybrid deep model
Ref (Hosny et al., 2023).	PlantVillage	CNN + LBP Fusion	Deep + handcrafted feature fusion	97.8	0.978	Increased complexity
Ref (Alhammad et al., 2025).	Potato Leaf Dataset	Explainable CNN	Explainable DL with Grad-CAM	98.2	0.982	Focus on interpretability
Ref (Sebastian et al., 2024).	PlantVillage	Vision Transformer	Linear projection layers in ViT	98.9	0.989	Transformer-optimized
CNN (This Work)	Augmented iBean	Custom CNN	Local feature extraction via convolution	97.01	0.97	Lightweight baseline model
ViT (This Work)	Augmented iBean	Vision Transformer	Self-attention & patch embeddings	98.18	0.98	Strong global pattern modeling
ResNet18 (This Work)	Augmented iBean	Residual CNN	Skip connections, hierarchical learning	99	0.99	Best accuracy-efficiency trade-off

**Table 5** presents a structured comparison of representative deep learning architectures for plant disease detection, highlighting their core architectural mechanisms and reported F1-score performance. Although the referenced studies employ different datasets and evaluation settings, the comparison serves as a contextual benchmark to position the present models within the broader research landscape. The results indicate that the ResNet18 architecture evaluated under standardized preprocessing and training conditions achieves competitive F1-score performance relative to transformer-based and hybrid approaches. This comparison underscores the effectiveness of controlled architectural benchmarking in analyzing trade-offs between model complexity, feature representation strategies, and classification performance.

**Table 5:** Comparative overview of F1-score performance and architectural characteristics across representative plant disease detection models.

Model	F1 score	Architecture aspect	Dataset
CNN (This Work)	0.97	Local convolutional feature extraction	Augmented iBean
ViT (This Work)	0.98	Patch embeddings + self-attention	Augmented iBean
ResNet18 (This Work)	0.99	Residual skip connections	Augmented iBean
ViTAL	0.989	Linear projections in ViT	PlantVillage
Hybrid CNN-ViT	0.985	CNN encoder + Transformer attention	PlantVillage
CNN + LBP Fusion	0.978	Deep + handcrafted feature fusion	PlantVillage
Explainable CNN	0.982	Explainable DL (Grad-CAM)	Potato Leaf

1. CNN Limitations: The standard CNN model, despite achieving a strong validation accuracy of 97.01%, shows clear limitations compared to deeper architectures like ResNet-18 and ViT. Unlike ResNet’s skip connections that enable deeper learning of complex, fine-grained patterns, the simpler CNN architecture is shallower and primarily focuses on local features, making it less effective at distinguishing visually similar diseases such as angular leaf spot and bean rust. Angular leaf spot and bean rust share similar local visual features (color, texture), but CNN processes the image with small kernels 3x3, 5x5) that only see neighboring pixels. It cannot simultaneously detect lesions scattered across multiple leaf regions. In contrast, ViT’s self-attention mechanism captures global contextual relationships across the entire image, allowing it to recognize subtle, long-range texture patterns that the CNN fails to model effectively.
2. Vision Transformer Strengths: The Vision Transformer (ViT) achieved an excellent 98.18% validation accuracy, showcasing strong global feature extraction through its self-attention mechanism. ViT divides your 224x224 leaf into 196 patches 14x14 grid) and uses self-attention to directly compare all patches with each other. This enables ViT to detect that Angular leaf spot shows clustered, progressive patterns along leaf veins, Bean rust shows random, distributed pustules across the surface. This global spatial distinction explains why ViT gets fewer misclassifications (85 vs CNN’s 137). However, in a few challenging cases, ViT struggled to distinguish angular leaf spot from bean rust due to their extreme visual similarity. Unlike ResNet-18’s hierarchical learning of low-level textures to high-level shapes, ViT’s global attention may have overlooked subtle local texture cues critical for this distinction. Additionally, ViTs are data-hungry, and limited examples of these ambiguous cases may have hindered their ability to learn such fine-grained differences as effectively as ResNet-18.
3. ResNet18 Superiority: The ResNet-18 model proved to be the most effective architecture, achieving the highest validation accuracy (99%) and precision (99.01%) among all tested models. Its superiority stems from the introduction of residual (skip) connections, which solve the vanishing gradient problem by allowing gradients to flow directly through the network, enabling deeper and more efficient learning of complex features. Unlike Vision Transformers (ViTs), which rely on large datasets and must learn spatial relationships from scratch, ResNet-18 benefits from a strong inductive bias—its inherent understanding of image structure through local feature extraction and hierarchical representation. This makes it highly data-efficient and particularly effective at capturing fine-grained visual cues, allowing it to outperform both the standard CNN and ViT in distinguishing subtle disease patterns on bean leaves.
4. Implications for Agriculture: The results of the research suggest that ResNet18 is the most pragmatic approach to deploy the model large scale for farms. CNN serves as the best baseline model in environments with bare minimum computational resources.

The overall conclusion of the research is that there are deep learning architectures such as ResNet18, and Vision Transformers that outdo CNN considerably in leaf disease classification tasks. This research shows that incorporating deep learning models with IoT enabled systems for real time monitoring of agriculture systems significantly helps with any disease outbreak elsewhere and improve sustainability and food security.

## 4 Conclusion

The study investigates the application of deep learning techniques for the classification of bean leaf diseases using a large-scale publicly available dataset. Three models are evaluated: a Convolutional Neural Network (CNN), a Vision Transformer (ViT), and ResNet18. Each model is analyzed based on its accuracy, precision, and ability to generalize across the three categories of interest: angular leaf spot, bean rust, and healthy bean leaves.

The experimental results demonstrate that CNN achieves only moderate performance, with a validation accuracy of 97%, though it exhibits limitations in distinguishing visually similar disease symptoms. While CNN can identify broad visual patterns, it struggles to distinguish fine-grained disease features, which often overlap in appearance. The Vision Transformer, in contrast, achieves superior performance, with validation accuracy of 98.18%. Due to its attention mechanism, ViT can draw global relationships across the leaf surface, leading to strong classification performance regardless of whether the visual conditions are challenging or not. However, ViT requires significant compute, so it is less appropriate for deployment on low-power or edge devices.

Overall, ResNet18 provided the best results out of the three models, with a validation accuracy of 99% and precision of 99.01%. The residual connections of the neural network allowed it to avoid the vanishing gradient problem and efficiently learn hierarchical representations of the disease patterns; in comparison, ResNet18, when compared to ViT, offered near-perfect trade-offs to achieve the level of accuracy and computational efficiency it provided, and is overall the most feasible to implement in real-world agricultural applications.

In terms of agricultural impact, these results are meaningful, as early and reliable disease detection can significantly reduce yield losses and improve disease management strategies, particularly for critical crops such as beans that are vital to global nutrition and food security. Automated deep learning-based systems, when coupled with IoT and edge-based platforms, can support timely disease intervention, reduce manual labor, and enhance precision-agriculture workflows.

This study contributes to the growing body of research on artificial intelligence in agriculture by offering a rigorous comparison of three influential model families and providing evidence of performance-efficiency trade-offs. The findings reinforce that while transformers are emerging as powerful tools for plant pathology, lightweight architectures such as ResNet18 currently offer the most realistic path to field deployment.

Future work will focus on three practical directions to enhance real-world applicability. First, the ResNet18 architecture will be optimized into a lightweight inference model for deployment on edge devices such as smartphones, drones, and embedded agricultural systems, with evaluation of latency, power consumption, and real-time performance. Second, the integration of the model within a complete IoT-based smart farming pipeline will be explored, enabling automated workflows from image acquisition to disease diagnosis and decision support. Third, the transferability of the trained model to different crops and disease categories will be investigated to assess its potential as a generalized plant disease recognition framework.

In conclusion, this research demonstrates that deep learning techniques represent an effective approach for automated bean leaf disease recognition. While CNNs form a strong baseline, both Vision Transformers and ResNet18 achieve significantly higher performance, with ResNet18 emerging as the most promising model for practical and scalable deployment in precision agriculture. These results highlight the transformative potential of artificial intelligence in improving crop health monitoring and supporting sustainable agriculture practices worldwide.

## Data Availability

Publicly available datasets were analyzed in this study. This data can be found here: https://ieee-dataport.org/documents/bean-leaf-disease-augmented-ibean-dataset.
